# Valence-programmable nanoparticle architectures

**DOI:** 10.1038/s41467-020-16157-0

**Published:** 2020-05-08

**Authors:** Sha Sun, Shize Yang, Huolin L. Xin, Dmytro Nykypanchuk, Mingzhao Liu, Honghu Zhang, Oleg Gang

**Affiliations:** 1 Department of Biochemistry and Molecular Biology, School of Basic Medical Sciences, Xi’an Jiaotong University Health Science Center, 710061 Xi’an, China; 2 Center for Functional Nanomaterials, Brookhaven National Laboratory, Upton, NY 11973 USA; 3 Department of Chemical Engineering and Department of Applied Physics and Applied Mathematics, Columbia University, New York, NY 10027 USA

**Keywords:** Nanoscale materials, Soft materials, Organizing materials with DNA, Organic-inorganic nanostructures

## Abstract

Nanoparticle-based clusters permit the harvesting of collective and emergent properties, with applications ranging from optics and sensing to information processing and catalysis. However, existing approaches to create such architectures are typically system-specific, which limits designability and fabrication. Our work addresses this challenge by demonstrating that cluster architectures can be rationally formed using components with programmable valence. We realize cluster assemblies by employing a three-dimensional (3D) DNA meshframe with high spatial symmetry as a site-programmable scaffold, which can be prescribed with desired valence modes and affinity types. Thus, this meshframe serves as a versatile platform for coordination of nanoparticles into desired cluster architectures. Using the same underlying frame, we show the realization of a variety of preprogrammed designed valence modes, which allows for assembling 3D clusters with complex architectures. The structures of assembled 3D clusters are verified by electron microcopy imaging, cryo-EM tomography and in-situ X-ray scattering methods. We also find a close agreement between structural and optical properties of designed chiral architectures.

## Abstract (web summary)

Assembling nanoparticles into precise architectures by controlling their positions in three-dimensional space is a major challenge in nanoscience. Here, the authors construct complex, preprogrammed clusters of DNA-encoded nanoparticles by coordinating them onto a DNA mesh wireframe.

## Introduction

Nanoparticle-based architectures have drawn much attention for decades due to their special electromagnetic^1,2^ and optical^1,3–5^ characteristics derived from the collective effect of building blocks. The properties of these architectures depend on the individual particle behavior, and more importantly, on the structural details of these architectures^6^. However, unlike molecular architectures whose complexity can be predicted and realized through valence and bond orientation of individual atoms, mimicking atomic valence for nanoparticles and utilizing it to build clusters is problematic due to the limitation in precise manipulation of nanoparticle surfaces and prescribing their 3D-binding characteristics. Thus, other strategies for creating desired nanoparticle architectures are considered.

For example, different types of clusters were observed for spherical micron-sized particles, where specific organizations are determined by packing effects^7,8^. It has also been shown that particle shapes can have a dramatic effect on phase behavior^9,10^. While considerable developments in synthesis of anisotropic nanoparticles^11^ have been made, the use of shapes for cluster engineering is challenging due to the complex interplay of entropic and interaction effects. For micron-sized colloids, several successful approaches for building designed clusters were shown using particle shape complementarity^12,13^, colloidal fusion^14^, or specifically located interaction patches formed by deposition^15–17^ or DNA methods^18,19^. However, transferring these approaches to the nanoscale remains a challenge.

Two conceptually different strategies for cluster assembly might be considered at the nanoscale. One idea is to form a desired cluster by introducing molecular information on nanoparticle surfaces via printing^20,21^ or wrapping^22,23^ by prescribed DNA nanostructures, forming patches with nanoscale resolution. The other idea to create clusters is by coordinating nanoparticles using a certain multilinking block with a well-defined valence mode. This approach allowed for assembling clusters and lattices using an anisotropic nanoparticle that locks the arrangement of spherical nanoparticles^24^. The thriving development of structural DNA nanotechnology, especially DNA origami^25–30^, which is formed by folding a long viral DNA with the help of short staple strands, offers a powerful solution for coordinating particles with designed DNA constructs. Such DNA constructs have been exploited to assemble nanoparticles into clusters^31–34^, periodic one-dimensional (1D)^22,32,35^, two-dimensional (2D)^22,32,36^ and three-dimensional (3D)^37–39^ organizations. The problem, however, is that the specific shape of a given DNA construct sets a cluster architecture; thus, formation of a different desired architecture requires a use of a different, specifically fabricated DNA construct with a designated shape.

**Fig. 1:**
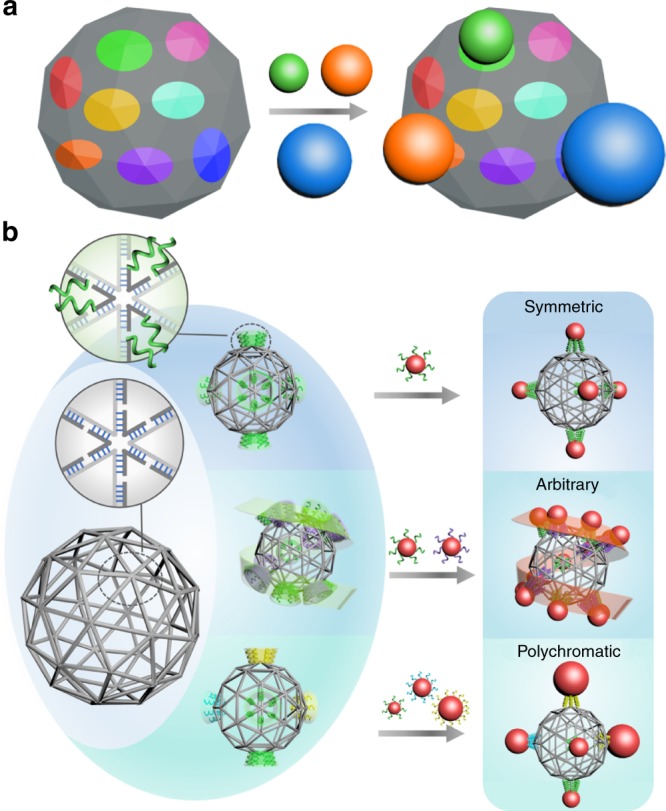
Nanoparticle-cluster self-assembly directed by the valence-programmable DNA meshframe. **a** Conceptual illustration of a nanoparticle (NP) cluster coordinated by sphere-like frame structure with arbitrarily prescribed valence modes and different types of binding affinities (shown as colors). Designated vertices provide binding affinities to corresponding DNA-encoded NPs (shown with matched colors). **b** Designed DNA meshframe origami, pentakis icosidodecahedron (gray skeleton), for programming designed NP cluster architectures. Zoomed-in vertex shows that it is formed by six edges, with each edge consisting of one double helix. Dark gray lines indicate staple strands and light gray lines indicate a templating DNA. The DNA meshframe can be encoded by introducing ssDNA around the vertex. An encoded vertex is zoomed-in to show that six identical sticky ends (green curves) protrude from a designated vertex. Sticky ends anchored on designated vertices form valence modes of triangular bipyramid and helix (top and middle). Distinctive sets of sticky ends (strands with different colors) can be anchored at designated vertices (middle and bottom). NPs (red balls), capped with complementary DNA shells, are assembled into designed clusters through their coordination around the meshframe corresponding with the programmed vertices, for example (from top to bottom): symmetric nanocluster, arbitrarily prescribed nanocluster with chiral helical valence mode, and multitype NP cluster.

Here, we propose the idea of using a sphere-like DNA mesh structure (Fig. 1a) as a highly symmetric frame, which can be programmed to exhibit different prescribed valence modes, and consequently, used to coordinate nanoparticles into pre-defined architectures. Moreover, specific positions and types of bonds can be fully prescribed. This approach offers designability over different valence modes using the same underlying high-symmetry frame, including various subset symmetries, arbitrarily prescribed helix-like valence, and valence with different types of affinities. Accordingly, a variety of cluster architectures with single-type and multitype nanoparticles can be rationally formed. Furthermore, we show that using planar and spatial imaging and in situ scattering methods, such programmable frames coordinate nanoparticles into a variety of corresponding cluster architectures possessing symmetric, helical, and site-specific nanoparticle organizations.

## Results

### Design of valence-programmable nanoparticle clusters

We first discuss how to program a desired nanoparticle (NP) cluster architecture using a DNA scaffold and then what limitations exist when using this strategy for cluster assembly. It is highly advantageous to program the underlying scaffold to be capable of exhibiting desired valence. Moreover, such valence does not have to be limited to symmetry modes found in atomic systems, but it can address a wider space due to the ability to fully prescribe bond locations and types. Although, in general, DNA-binding sites can be placed in desired locations on the DNA scaffold, the specific design and geometrical limitations of the scaffold become restricting factors very quickly. Indeed, the overall shape of the DNA construct will have a major impact on the nanoparticle-cluster architecture. In order to minimize any effect arising from DNA-scaffold shape anisotropy, such a scaffold should possess the highest symmetry; in other words, it should be designed to be as close as possible to a sphere. At the same time, it should be able to provide binding sites for nanoparticles around this topology. Ideally, such objects should have the capability for bond programming in order to afford different types of valence modes through the 3D placement of specific binding sites on this object. Unlike atomic systems, different sites can be distinguished through orthogonal DNA-encoding, and this so-called polychromatic valence^22,40^ offers distinctive bonds for DNA-encoded NP bindings. Such desired programmable DNA object can address the challenge of creating a designed cluster, and we illustrate this concept for such assembly in Fig. 1a.

To realize the idea of universal linking frame experimentally, we used DNA origami to fabricate a sphere-like mesh construct^28^, which has a pentakis icosidodecahedron shape (Fig. 1b). Icosahedral symmetry (*I*_*h*_) is the most common symmetry for polyhedrons approximating a sphere. This structure contains 42 vertices, with each vertex being a junction of either five or six edges. 90% of the edges (108 of 120) contain one DNA double helix and the other 10% contain two DNA double helices, with an average length of ~15.7 nm (~47 base pairs (bp), assuming 3.5 nm/10.5 bp for DNA double helix). For this highly symmetrical object, a desired vertex, surrounded either by six or eight helices, can be used as a binding site for NPs by encoding six identical single-stranded DNA (ssDNA) around it, i.e., incorporating so-called “sticky ends” for NP binding. We designed such sticky ends with a 2-base inner spacer region and 11-base outer recognition region. By choosing and encoding desired subgroups of vertices on a sphere-like meshframe, we program a desired valence mode while maintaining the shape of the underlying DNA scaffold. Subsequently, DNA-capped NPs can bind to those designated sites and form designed cluster architectures. Furthermore, polychromatic valence can be generated by encoding the meshframe with different types, or sequence specificities, of sticky ends to recognize different correspondingly encoded NPs. Using this method, we can achieve NP assemblies with symmetric valence, such as a five-fold cluster, nanoclusters with arbitrary valence, such as a spherical helix cluster, and multitype nanoclusters, such as a three-component chiral cluster, as demonstrated in the conceptual Fig. 1b.

### Symmetric architectures

**Fig. 2:**
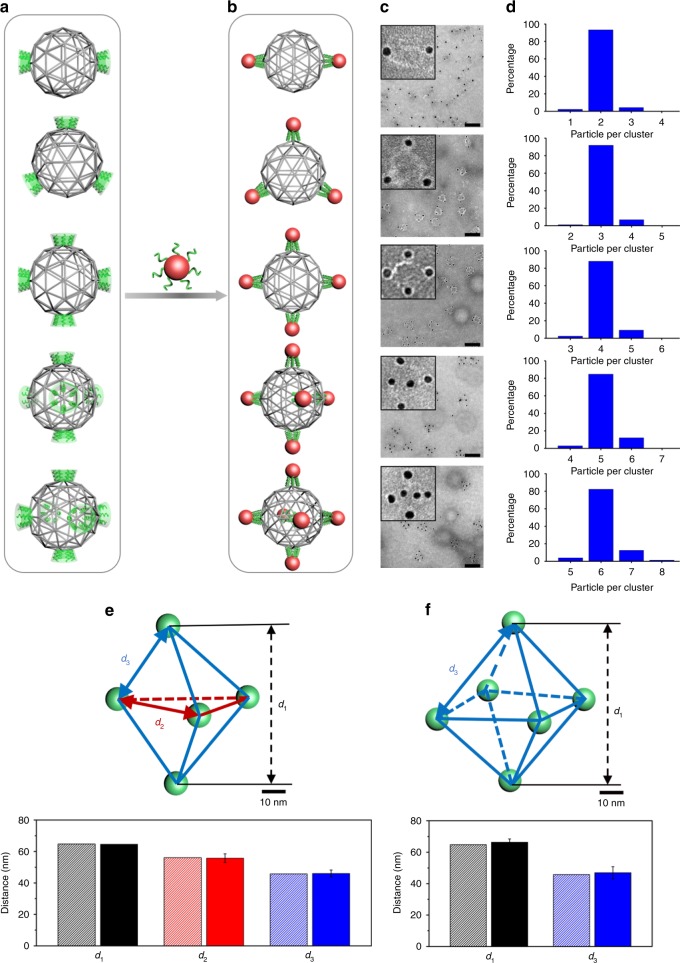
Structures of NP cluster architectures assembled by programmed meshframe and revealed by TEM and cryo-TEM tomography. **a** (From top to bottom) Designed DNA meshframes with different valence numbers: two, three, four, five, and six, corresponding to the geometry of dumbbell, triangle, square, TBP and octahedra, respectively. **b** NPs decorated with DNA are assembled into clusters, whose architecture is determined by the meshframe valence mode. **c** Representative negative-stained TEM images of assembled NP clusters based on meshframes with different valence modes; scale bar, 100 nm (insets: zoomed-in images; side length, 70 nm). **d** NP cluster population histograms. **e** Reconstructed 3D structure of TBP cluster from cryo-TEM based tomography (top). Designed center-to-center distances between NPs (bottom, shadow columns): *d*_*1*_ = 64.8 nm, *d*_*2*_ = 56.1 nm, and *d*_*3*_ = 45.8 nm. Measured distances between NPs of one reconstructed cluster (bottom, solid columns): *d*_*1*_ = 64.6 nm, *d*_*2*_ = 55.8 ± 2.7 nm, and *d*_*3*_ = 46.1 ± 2.1 nm. **f** Reconstructed 3D structure of octahedral cluster by cryo-TEM tomography (top). Designed NP distances (bottom, shadow columns): *d*_*1*_ = 64.8 nm and *d*_*3*_ = 45.8 nm, and measured cryo-TEM tomography distances averaged over two independently reconstructed clusters (bottom, solid columns): *d*_*1*_ = 66.3 ± 2.0 nm and *d*_*3*_ = 46.9 ± 3.8 nm. Error bars indicate standard deviation of experimental data.

First, to explore the valence-programmability of the DNA sphere-like meshframe, we designed a class of frames with various symmetric valence modes, from two to six, corresponding to the geometries of dumbbell, triangle, square, triangular bipyramid (TBP), and octahedra, respectively (Fig. 2a, see more design details in Supplementary Discussion, Design of valency modes on the sphere-like DNA meshframe). NPs functionalized with DNA can bind through hybridization to complementary-encoded sites, resulting in the assembly of matching clusters with designed architectures (Fig. 2b).

To fabricate the programmable meshframes, we annealed M13mp18 scaffold with a specific set of staple strands (Supplementary Fig. 7, see Supplementary Table 2 and 3 for sequence information). Then, the preassembled frames were mixed and annealed with gold NPs (AuNPs, 10 nm core diameter) to construct NP clusters, followed by purification with agarose gel electrophoresis (see Methods section for NP cluster assembly and purification details). Representative TEM images demonstrate the projections of five types of nanoclusters, consistent with corresponding designs (Fig. 2c, Supplementary Figs. 9–13). We note that in comparison with AuNPs, stained meshframe edges formed by only one or two duplexes, have a weak contrast. We have observed different projections of these clusters, with some clusters being deformed on a TEM grid due to the limited rigidity of meshframe that could not withstand drying process during a TEM sample preparation. The statistical analysis shows a high yield for all assembled types of the cluster architectures: ~90% of dumbbell, triangular and square nanoclusters show the correct number and positions of NPs (of ~500 for each type of cluster), and >80% for TBP and octahedral nanoclusters exhibit expected morphology (of ~400 for each type of cluster) (Fig. 2d). TEM statistics also shows that ~99% of binding sites on the meshframe are occupied by NPs. A high NP attachment yield per binding site owes to the rational design of sticky-end distribution and binding strength. Among the occupied sites, 98% are specifically bound by individual particles as designed, and 2% are shared by two particles. We assume that the double occupancy phenomenon results from non-equilibrium hybridization, where a second NP binds before all sticky ends at a given site can connect with the first NP. This effect of double occupancy is reduced for larger NPs. For example, clusters assembled with 20 nm AuNPs show 99% meshframe site occupancy by individual NPs and that only 1% of sites are shared by two NPs (Supplementary Fig. 15).

To reveal the spatial arrangement of NPs in the assembled 3D nanoclusters, such as TBP and octahedra arrangement, we employed cryo-EM based tomography (Supplementary Figs. 25 and 26). Fig. 2e shows that the five NPs of the reconstructed TBP cluster are arranged in a designed manner. To quantify TBP cluster structural parameters, we measured distances between NPs on the diagonal (one *d*_*1*_), on the horizonal triangular plane (three *d*_*2*_) and on the lateral plane (six *d*_*3*_). The distances agree well with designed values (see Supplementary Discussion, Theoretical interparticle distances of symmetric nanoclusters), indicating the precise, successful arrangement of NPs on the sphere as shown in Fig. 2e, bottom. Similarly, tomographic measurement, and reconstruction (Supplementary Fig. 26) were carried out for an octahedral nanocluster, and the results also demonstrate (Fig. 2f) an excellent correspondence between the targeted and realized architectures of the interparticle distances (three *d*_*1*_, twelve *d*_*3*_).

**Fig. 3:**
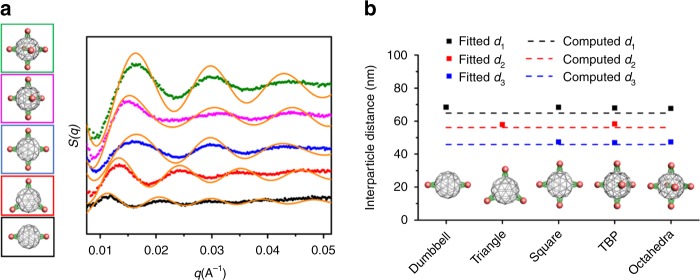
In-liquid structures of NP cluster architectures revealed by in situ SAXS. **a** Extracted structure factor S(*q*) from in situ SAXS for designed clusters with the following meshframe valence numbers (from bottom to top): two (black), three (red), four (blue), five (magenta), and six (green), respectively. Fitted S(*q*), as discussed in the text, are shown by orange curves. **b** Interparticle distances derived from SAXS (squares) for NP clusters: *d*_*1*_ = 68.4 nm for dumbbell cluster, *d*_*2*_ = 58.0 nm for triangular cluster, *d*_*1*_ = 68.3 nm and *d*_*3*_ = 47.4 nm for square cluster, *d*_*1*_ = 67.9 nm, *d*_*2*_ = 58.2 nm and *d*_*3*_ = 46.8 nm for TBP cluster, *d*_*1*_ = 67.6 nm and *d*_*3*_ = 47.3 nm for octahedral cluster. Interparticle distances computed for the designed architectures (dash lines): *d*_*1*_ = 64.8 nm, *d*_*2*_ = 56.1 nm, and *d*_*3*_ = 45.8 nm.

To probe the structures of formed clusters in the actual buffer conditions in which the clusters were assembled, we employed in situ synchrotron-based small-angle X-ray scattering (SAXS). Purified nanoclusters, dispersed in solution, were placed in a quartz capillary, and then probed at room temperature by a collimated X-ray beam (λ = 0.92 Å) at the Complex Materials Scattering (CMS) beamline of National Synchrotron Light Source II (NSLS-II) at Brookhaven National Laboratory. The scattering pattern was collected with a Dectris Pilatus3 X 2 M pixel-array detector and converted to 1D scattering intensity versus wavevector transfer, *q*. Structure factor, *S*(*q*), for each nanocluster was extracted from the 1D scattering intensity by subtracting background and dividing the intensity by the form factor of AuNPs^41,42^ (Fig. 3a), which was measured for dispersed AuNPs. Interparticle center-to-center distances are derived by fitting *S*(*q*) with the function below:

1 $${\it{S}}\left( {\it{q}} \right) = 1 + \frac{2}{{\it{N}}}\mathop {\sum}\limits_{\scriptstyle j = 1\hfill \atop\\ \scriptstyle k > j\hfill }^{\it{N}} {\frac{{{\it{\sin}}\big( {{\it{qd}}_{{\it{jk}}}} \big)}}{{{\it{qd}}_{{\it{jk}}}}}}$$

where *N* is the NP number in a specific designed cluster, and *d*_*jk*_ is the center-to-center distance for each pair of NPs in a cluster. For dumbbell and triangular nanoclusters, only one type of interparticle distance exists, which is *d*_*1*_ between diagonal nanoparticles in the dumbbell nanocluster and *d*_*2*_ between nanoparticles on the triangular plane in the triangular nanocluster. For the square nanocluster, two types of interparticle distances, *d*_*1*_ between diagonal nanoparticles and *d*_*3*_ between adjacent nanoparticles, exist. The average interparticle distances for five-cluster architectures are *d*_*1*_ = 68.1 ± 0.3 nm, *d*_*2*_ = 58.1 ± 0.1 nm, and *d*_*3*_ = 47.2 ± 0.3 nm, respectively, which are close to the expected values based on the design: *d*_*1*_ = 64.8 nm, *d*_*2*_ = 56.1 nm, and *d*_*3*_ = 45.8 nm (Fig. 3b, see details in Supplementary Discussion, Interparticle distances of nanoclusters fitted from SAXS).

### Arbitrarily designed cluster: spherical helix architecture

**Fig. 4:**
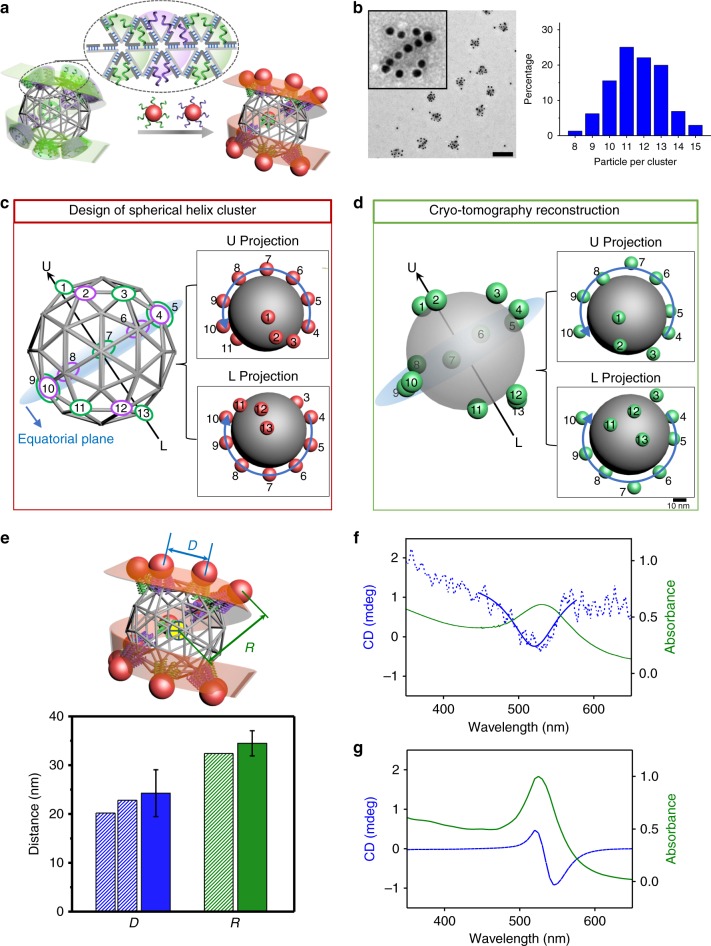
Design and structural and optical characterization of the spherical helix NP cluster. **a** Schematic of the spherical helix NP cluster. The spherical helix valence mode is realized for the meshframe by programming two alternating sets of sticky ends (green and purple, with five strands for each color) at desired vertices. Two types of AuNPs with identical 10 nm core size but distinct DNA shells (green and purple) are directed through the hybridization to binding sites and assembled into a spherical helix cluster. **b** (Left) Representative negative-stained TEM image of spherical helix clusters; scale bar, 200 nm (inset: zoomed-in image of a spherical helix cluster; side length, 150 nm). (Right) Population analysis of assembled spherical helix clusters (from 611 measured clusters). **c** Designed NP positions of the spherical helix cluster (numbered from 1 to 13) on a sphere-like meshframe (left). Seven particles (Particles 4–10) are on the equatorial plane of the sphere-like structure. Particle 1 and Particle 13 denote the summits of Upper and Lower hemispheres, respectively. Projections of designed spherical helix cluster from the Upper (upper right) and Lower (lower right) summits. The grey sphere provides visual guidance. The blue line with arrow defines particles on the equatorial plane (Particles 4–10). **d** Spherical helix cluster reconstructed from cryo-TEM tomography. 3D view of the reconstructed structure (left) and projections from the Upper (upper right) and Lower (lower right) summits. **e** Center-to-center distance between adjacent NPs, *D*, and center-to-center distance between sphere-like meshframe and NPs, *R*, were measured. Designed distances (bottom histogram, shadow columns): *D* = 20.2 nm and 22.8 nm, and *R* = 32.4 nm. Average distances obtained from three independently reconstructed clusters (bottom histogram, solid columns): *D* = 24.0 ± 4.9 nm and *R* = 34.5 ± 3.8 nm. Error bars indicate standard deviation of experimental data. **f** Computed CD spectrum (blue curve) and absorption spectrum (green curve) for spherical helix clusters. **g** Experimental CD spectrum (blue dotted curve), Lorentzian fit (blue solid curve) and absorption spectrum (green curve) for spherical helix clusters as described in the text.

To demonstrate the versatility and flexibility of this assembly strategy for creating complex and arbitrarily designed clusters, we implemented an architecture in which NPs are located in a helical pattern on the surface of sphere-like DNA object (Fig. 4). We programmed our DNA meshframe to have 13 binding sites at designated vertices, encoding a left-handed spherical helix cluster formed by 13 NPs (Fig. 4a). Two different sets of sticky ends, placed in alternating order between the sites, with five identical sequences per site (see Supplementary Table 4 for DNA sequence information), are used. By alternating these two distinctive sets of sticky ends, we can reduce site competition between neighboring NPs and thus increase the accuracy of NP localization on the designated vertices. Accordingly, two types of 10 nm AuNPs decorated with different DNA shells are used to bind with the helically positioned binding sites. We note that for this high valence DNA scaffold, an excess of DNA staple strands used in scaffold formation has to be removed before mixing with NPs, in order to minimize inter-cluster crosslinking. The mixtures of purified meshframes and AuNPs were annealed from 37 °C to 20 °C for 12 h, followed by purification with agarose gel electrophoresis and analysis with negative-stained TEM. Representative TEM images show that the NP cluster matches the designed helical morphology (Fig. 4b, left, Supplementary Fig. 18), with 88.4% of binding sites occupied by NPs (Fig. 4b, right), as measured from 611 clusters. This yield is lower than that of symmetric clusters discussed above (~99%), due to more complex NP arrangements in a helical pattern and stronger steric interactions between adjacent DNA-capped AuNPs.

To unravel information regarding particle organization for the spherical helix valence mode on the meshframe, we applied cryo-TEM-based tomography to characterize the 3D structure of individual clusters (Supplementary Fig. 27). To establish a comparison with a prescribed helical valence arrangement, we numbered the particle sites on the sphere-like meshframe from 1 to 13 (Fig. 4c, left). Seven particles (Particle 4–10) are located on the equatorial plane of the sphere. Particle 1 and Particle 13 are located on the summits of Upper (U) and Lower (L) hemispheres, respectively. We projected the structure from both the U and L summits. For visual guidance, in the center of the cluster we added a gray sphere, whose radius is equal to the distance between the meshframe center and NP surface. Both projections show a uniform NP arrangement on the equatorial plane and NP positions on the upper and lower hemispheres (Fig. 4c, right).

The reconstructed nanocluster is presented in both 3D view and 2D projections in Fig. 4d, where a gray sphere provides visual guidance as Fig. 4c. The coordinate of sphere center and sphere radius are fitted using the obtained 3D coordinates of all NPs (numbered 1–13) in the reconstructed cluster (Supplementary Fig. 27). The tomography results (Fig. 4d, left) show that seven NPs (Particles 4–10) are on the equatorial plane, three NPs (Particle 1–3) on the upper sphere, and three (Particles 11–13) on the lower sphere. The visualized 3D spherical helix pattern of NPs matches well with the designed helical cluster architecture. The U and L projections (Fig. 4d, right) demonstrate that Particles 4–10 are arranged relatively uniformly on the equatorial plane. The locations of Particles 1–3 and Particles 11–13 indicate that their placement pattern corresponds to a left-handed chirality, which is in agreement with the prescribed valence mode. Small NP shifts (~5 nm) from expected positions are observed in the reconstructed structure, which might result from interparticle repulsion and sample preparation for imaging. We also note that since each binding site has multiple sticky ends, NPs can hybridize at slightly different configurations at the same site, which affects the precision of a NP placement. Although there are twelve values of interparticle center-to-center distances (*D*) for clusters with thirteen NPs (Fig. 4e), there are only two different *D* in our designed locations of NPs due to two types of edge lengths of the meshframe: ten *D* values are of 20.2 nm and the other two of 22.8 nm. Since the diameter of 10 nm AuNPs with DNA shells is ~20 nm (21 nucleotide (nt) for the DNA shell), which is close to the designed *D* value, steric repulsion may result in some displacement of NPs. Our tomographic measurements show only a small increase of *D* (24.0 ± 4.9 nm) over its two design values, as averaged from three independently reconstructed clusters (Supplementary Fig. 28), which supports the repulsion hypothesis. Such an increase of *D* is consistent with our SAXS measurements, which show a corresponding shift of the primary scattering peak that arises from nearest neighbor interparticle distances (Supplementary Fig. 4). Another obtained value in this experiment is the distance between the center of meshframe and the center of individual AuNP in the nanocluster (Fig. 4e), *R* = 34.5 ± 3.8 nm, which matches well the expected value (*d*_*1*_/2 = 32.4 nm) and our SAXS measurements discussed above (68.1/2 = 34.0 nm). The SAXS measurements for spherical helical clusters provide only a coarse information about internal cluster organization due to the sensitivity of a structure factor to an interparticle distance distribution. The detailed analysis indicates that the design of clusters with a close proximity of nanoparticles might increase a distribution of interparticle distances in comparison to symmetrical clusters (Supplementary Discussion, SAXS analysis and theoretical calculations of spherical helix clusters).

In solution, the optical absorption spectrum of spherical helix clusters, with characteristic absorption peak at 520 nm (Supplementary Fig. 17), closely resembled the surface plasmon resonance (SPR) mode of individual AuNPs, which indicates a weak coupling between NPs. Due to the chiral nature of the helical cluster of plasmonic NPs, a different absorption for incident light of left and right circular polarization can lead to a circular dichroism (CD) signature near the SPR. To enhance the plasmonic resonance through interparticle coupling, we increase NP size to ~13 nm using gold enhancement reagent^43^; this also decreases interparticle distances accordingly. A corresponding plasmonic redshift from 520 nm to 532 nm (Fig. 4f) was observed. A positive CD signal was detected at wavelength shorter than 500 nm, with a negative dip around the SPR peak. The observation is consistent with our numerical simulation of the absorption and CD spectra of AuNP spherical helix clusters (Fig. 4g), using discrete dipole approximation (DDA)^44^ (see Supplementary Methods for simulation details). We note that this type of cluster combines a helical arrangement of NPs with overall spherical shape, and that compact and symmetric design is distinct from previously reported bar-like clusters with a spiral NP arrangement^31^.

### Multitype nanoparticles clusters

**Fig. 5:**
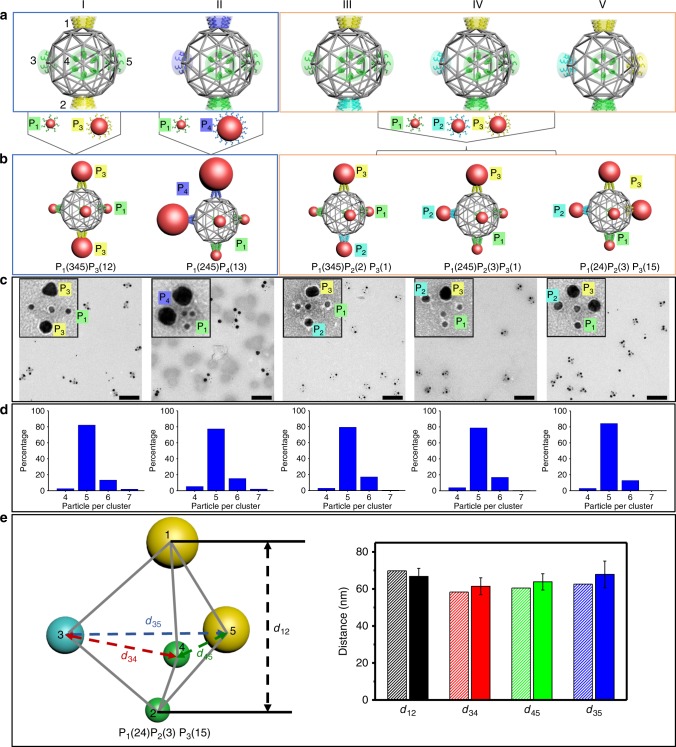
Design and characterization of multitype NPs clusters assembled with a meshframe of directional and polychromatic valence modes. **a** Meshframe programmed with a valence of TBP geometry and encoded ‘colored’ sites at selected vertices, labelled from 1 to 5, results in polychromatic valence. (I–II) two-color valence. (III–V) three-color valence. **b** Multitype NP clusters. (I) P_1_(345)P_3_(12) with three P_1_ (10 nm AuNP) at sites 3–4–5 and two P_3_ (20 nm AuNP) on sites 1–2; (II) P_1_(245)P_4_(13) with three P_1_ at sites 2–4–5 and two P_4_ (30 nm AuNP) at sites 1–3; (III) P_1_(345) P_2_(2)P_3_(1) with three P_1_ at sites 3–4–5, one P_2_ (15 nm AuNP) at site 2 and one P_3_ at site 1; (IV) P_1_(245) P_2_(3)P_3_(1) with three P_1_ at sites 2-4-5, one P_2_ at site 3 and one P_3_ at site 1; (V) P_1_(24) P_2_(3)P_3_(15) with two P_1_ at sites 2–4, one P_2_ at site 3 and two P_3_ at sites 1–5. **c** Representative negative-stained TEM images of NP hetero-clusters; scale bar, 200 nm (insets: zoomed-in images; side length, 100 nm). **d** Population histograms for corresponding multitype NP clusters. **e** Cryo-TEM tomography reconstructed P_1_(24)P_2_(3)P_3_(15) cluster (left). Designed interparticle distances (right, shadow columns): *d*_*12*_ = 69.8 nm, *d*_*34*_ = 58.3 nm, *d*_*45*_ = 60.5 nm, and *d*_*35*_ = 62.6 nm. Measured distances, as averaged over eight independently reconstructed P_1_(24)P_2_(3)P_3_(15) clusters (right, solid columns): *d*_*12*_ = 66.8 ± 4.3 nm, *d*_*34*_ = 61.4 ± 4.6 nm, *d*_*45*_ = 63.8 ± 4.4 nm, and *d*_*35*_ = 67.8 ± 7.2 nm. Error bars indicate standard deviation of experimental data.

The addressability of the sphere-like DNA meshframe not only allows prescribing directional valence through the location of binding sites, but also encoding each site independently such that it provides affinity only to the NP with matching encoding, as determined by the complementarity between sticky ends at the vertex and ssDNA in a given NP shell. We demonstrate that this encoded, so-called polychromatic valence opens opportunities for assembly of multitype NP cluster architectures (Fig. 5). As a demonstration of this capability, we program a meshframe with two- (Fig. 5a, I–II) and three-color valence modes (Fig. 5a, III–V) for assembly of different multitype NP clusters based on the same five-fold directional valence (Fig. 5b) discussed previously. Specifically, the binding sites of a TBP linking frame (labelled 1–5, Fig. 5a) are encoded independently with two and three sets of sticky ends (see Supplementary Table 5 for DNA sequence information). AuNPs with diameters of 10 nm (P_1_), 15 nm (P_2_), 20 nm (P_3_), and 30 nm (P_4_) were coated with specific “colored” DNA shells that are complementary to respective sticky ends on the meshframe. Guided by this polychromatic valence, different types of AuNPs were anchored to prescribed sites.

Fig. 5b (I) shows a two-component nanocluster with three-fold symmetry, with two P_3_ particles located at sites 1 and 2 and three P_1_ particles at sites 3, 4, and 5, providing a label of P_1_(345)P_3_(12). Similarly, a nonsymmetric two-component nanocluster P_1_(245)P_4_(13) was designed and assembled (Fig. 5b (II)). Then, a three-color valence meshframe was used to form symmetric and nonsymmetric nanoclusters: P_1_(345)P_2_(2)P_3_(1) with three-fold symmetry (Fig. 5b (III)), nonsymmetric achiral P_1_(245)P_2_(3)P_3_(1) (Fig. 5b (IV)) and nonsymmetric chiral P_1_(24)P_2_(3)P_3_(15) (Fig. 5b (V)). Representative TEM images show that the NP arrangements for all five hetero-clusters based on the sphere-like meshframe with polychromatic valence agree well with our design (Fig. 5c, Supplementary Figs. 20–24). The statistical analysis indicates that this hetero-cluster assembly process can be realized with relatively high yield, with ~80% of correct NP composition (out of ~420 clusters for each cluster type) (Fig. 5d).

To obtain information about spatial arrangement of NPs in multitype NP clusters, we studied the most complex, nonsymmetric chiral cluster (P_1_(24)P_2_(3)P_3_(15)) using cryo-TEM tomography (Supplementary Fig. 29). As we show in Fig. 5e, the reconstruction confirms that three types of AuNPs are arranged in our designed manner, with two P_3_ particles at vertices 1 and 5, one P_2_ particle at vertex 3 and two P_1_ particles at vertices 2 and 4. We obtained interparticle distances from eight reconstructed nanoclusters (Supplementary Fig. 30). The center-to-center distances between diagonal NPs (*d*_*12*_) and between NPs on the horizonal triangular plane (*d*_*34*_, *d*_*45*_, and *d*_*35*_) are in excellent agreement with the designed values (Fig. 5e, right).

Here we demonstrated a methodology for assembly of designed nanopartciles clusters using valence-programmable DNA meshframe with 3D control of NP positions and incorporation of different types of NPs. Spatially and type-defined (polychromatic) valence modes can be rationally designed and programmed using a highly symmetric, sphere-like frame that serves as a universal 3D scaffold for coordinating NPs in designed 3D patterns. We showed that nanoclusters with symmetric and arbitrary valence modes can be created with high yield and high structural fidelity, including different prescribed symmetries and helical organizations. We also demonstrated that the concept of polychromatic valence permits assembly of multitype NP clusters. The quantitative agreement between the designed and assembled structures was demonstrated using a combination of TEM, tomography and X-ray scattering methods. A demonstrated, broadly applicable valence-programmable assembly strategy opens new routes for the rational fabrication of NP architectures via self-assembly, with customized architectures, compositions, and function.

## Methods

### Self-assembly of DNA meshframe

The sequences of staple strands were designed by vHelix^28^. Staple strands (Integrated DNA Technologies) and M13mp18 scaffold (Bayou Biolabs) were mixed in 0.5 × TE buffer (5 mM Tris, 1 mM EDTA, pH 8.0, supplemented with 10 mM MgCl_2_). The solution was annealed from 80 °C to 60 °C at a cooling rate of 1 min/°C and from 60 °C to 20 °C at a rate of 23 min/°C.

### DNA modification of gold nanoparticles

The thiolated DNA strands (HPLC, Integrated DNA Technologies, see Supplementary Table 6 for DNA sequence information) were first reduced by tris(2-carboxyethyl) phosphine (TCEP) solution (Sigma–Aldrich) with a molar ratio of 1:100 in water at 20 °C. After the incubation for 1.5 h, the thiolated DNA strands were purified by removing small molecules with MicroSpin G-25 columns (GE Healthcare). Then the purified thiolated DNA strands were mixed with aqueous spherical gold nanoparticle (AuNP) solution (Ted Pella) with a ratio of 300:1 for 10 nm AuNPs, 700:1 for 15 nm AuNPs, 1000:1 for 20 nm AuNPs, and 2100:1 for 30 nm AuNPs. After 2 h of incubation at 20 °C, 10 × phosphate-buffered saline (PBS) (100 mM, pH 7.4) was added to bring the final solution to be 1 × PBS (10 mM, pH 7.4). For 15 nm, 20 nm, and 30 nm AuNPs, 10% SDS was added to bring the final concentration to 0.01% SDS. After another 2 h of incubation, stepwise addition of salting buffer (1 × PBS buffer with 2 M sodium chloride) increased the concentration of the sodium chloride to 0.3 M. The solution was allowed to age for 12 h. To remove excess thiolated DNA strands, DNA-AuNP conjugates were washed four times by centrifuge. The supernatant was removed, and the fresh washing buffer (1 × PBS buffer with 100 mM sodium chloride) was used to rinse and disperse the DNA-AuNP conjugates. The purified DNA-AuNP conjugates were quantified by measuring the absorbance at 520 nm on PerkinElmer Lambda 25 spectrophotometer.

### Assembly and purification of AuNP clusters

DNA meshframe was mixed with AuNPs with the ratio of 3 *N*:1 (*N* is the valency number) in 0.5 × TE (supplemented with 10 mM MgCl_2_) and annealed from 50 °C to 20 °C for 12 h. The annealed samples and 1 kb DNA molecular weight marker (New England Biolabs) were loaded to a native 1.5% agarose gel with 0.5 × SYBR Gold (running buffer: 0.5 × TBE buffer, containing 44.5 mM Tris, 44.5 mM boric acid, and 1 mM EDTA, supplemented with 11 mM MgCl_2_) and gel electrophoresis was performed at 60 volts for 3 h in an ice bath. Target bands were excised and cut into small pieces. The gel pieces were placed into Freeze ‘N Squeeze columns (Bio-Rad Laboratories) and centrifuged at 3000 × *g* for 5 min to obtain purified AuNP clusters. For spherical helix clusters, DNA meshframe was first purified with agarose gel electrophoresis as described above and quantified by measuring the absorbance at 260 nm on PerkinElmer Lambda 35 spectrophotometer. Then, purified DNA meshframe was mixed with AuNPs with the ratio of 3 *N*:1 and annealed from 37 °C to 20 °C for 12 h. Finally, the annealed sample was purified with agarose gel electrophoresis as described above (see Supplementary Figs. 8, 14, 16, and 19 for gel electrophoresis images of clusters).

### Negative-stained TEM

Three microliter of sample was loaded on the glow-discharged, carbon-coated grid (300 mesh, Ted Pella) for 1 min, and the excess sample was removed by a piece of filter paper. Next, the grid was incubated in 2% uranyl acetate aqueous solution for 30 s, followed by using a piece of filter paper to dry it. TEM imaging was performed on a JEOL 1400 at 120 kV.

### Cryogenic electron tomography

Copper mesh grids (Carbon Film 300 mesh, Copper, Ted Pella) were held for 10 s and glow discharged for 20 s. The cryogenic sample was then prepared using the FEI Vitrobot with typical parameters of 3 μL sample, temperature at 4 °C, force at 0, humidity at 100%, wait time 4 s, and blot time 5 s. The as-prepared sample was transferred to a liquid nitrogen tank to be stored for later use. Single tilt cryogenic tomography holder (Gatan 626) was cooled down to below 90 K under liquid nitrogen before the sample was loaded. The holder was then inserted into the JEOL 1400 microscope to collect tomography image series under 120 keV from around −60 degrees to 60 degrees at a step of 10 degrees. The original images were first converted into a stack image using the ImageJ software. Contrast inversion, image alignment and tilt axis refinement were carried out in Tomviz manually. The refined image stack was further reconstructed into 3D volumes using the Simultaneous Iterative Reconstructive Technique (SIRT) algorithm imbedded in Tomviz. The reconstructed 3D volumes were then filtered and segmented in Avizo software to get the 3D center positions.

### Small-angle X-ray scattering (SAXS)

SAXS measurements were performed at BNL National Synchrotron Light Source II (NSLS-II) Complex Material Scattering (CMS) beamline. The purified samples were injected into glass capillary tubes for X-ray scattering experiments, which were performed under room temperature. Structure factors *S(q)*, where *q* is the wavevector, were obtained by the radial integration of 2D patterns and were divided by a nanoparticle form factor obtained from the scattering of solution-dispersed nanoparticles.

### Measurements of circular dichroism and optical absorption for spherical helix clusters

Purified spherical helix cluster was mixed with the Gold Enhancement reagent (Nanoprobes) with a volume ratio of 1:0.3 and incubated at room temperature for ~30 min.^43^ The circular dichroism and optical absorption of helical clusters were measured by a Jasco J-815 CD spectrometer.

## Supplementary information

Supplementary Information

## Data Availability

The data that support the findings of this study are available from the corresponding author upon reasonable request.
