# Supplementary material for: Valence-programmable nanoparticle architectures
Source: Nat Commun. 2020 May 8;11:2279. doi: 10.1038/s41467-020-16157-0 (PMC7210924; doi:10.1038/s41467-020-16157-0)
Supplement: Supplementary file 1 — Supplementary Information [file 41467_2020_16157_MOESM1_ESM.pdf]

Supplementary Information

Valence-Programmable Nanoparticles Architectures

S. Sha et al.

Supplementary Methods

Simulation of circular dichroism and optical absorption for spherical helix clusters. Circular dichroism and optical absorption spectra of spherical helix cluster were numerically calculated by discrete dipole approximation (DDA)[3], using the DDSCAT code by Draine and Flatau[3]. In the calculation, the DNA-AuNP cluster was treated as a helical chain of AuNPs immersed in a uniform dielectric medium of $\epsilon_m = 1.77$ (water). The 10 nm AuNPs were simplified as point dipoles with Mie polarizability $\alpha = 3V\,(\epsilon - \epsilon_m)/(\epsilon + 2\epsilon_m)$, where V was the volume of each AuNP and ϵ was the complex dielectric constant of gold[4]. The validity of such treatment stemmed from the very small size of the AuNPs, which had a radius R = 5 nm that was much shorter than both the optical wavelength and the interparticle distance (20.2 nm and 22.8 nm). Under these conditions, we could apply dipole approximation and safely neglect any higher order modes and their near field interactions. By calculating optical extinction cross-sections respectively under left and right circular polarized light (LCP and RCP) in the wavelength range of 300 – 800 nm, we obtained the circular dichroism spectrum $\sigma_{CD} = \sigma_{LCP} - \sigma_{RCP}$, and the polarization-averaged absorption spectrum $\sigma_{ext} = (\sigma_{LCP} + \sigma_{RCP})/2$. Due to the optical anisotropy of the helical cluster, we performed calculation for various orientations of the cluster with respect to the incident wavevector and presented the final spectra as ensemble averages. The orientation average followed the standard procedure of DDSCAT, in which we sampled 37 points for the angle between the helical axis and incident wavevector (θ) from 0° to 180°, with uniform sampling in $\cos\theta$, and 12 points for the rotation angle of the cluster around its helical axis (φ) from 0° to 360° , with uniform sampling in φ, for a total sampling of 444 orientations.

Supplementary Discussion

Design of valency modes on the sphere-like DNA meshframe

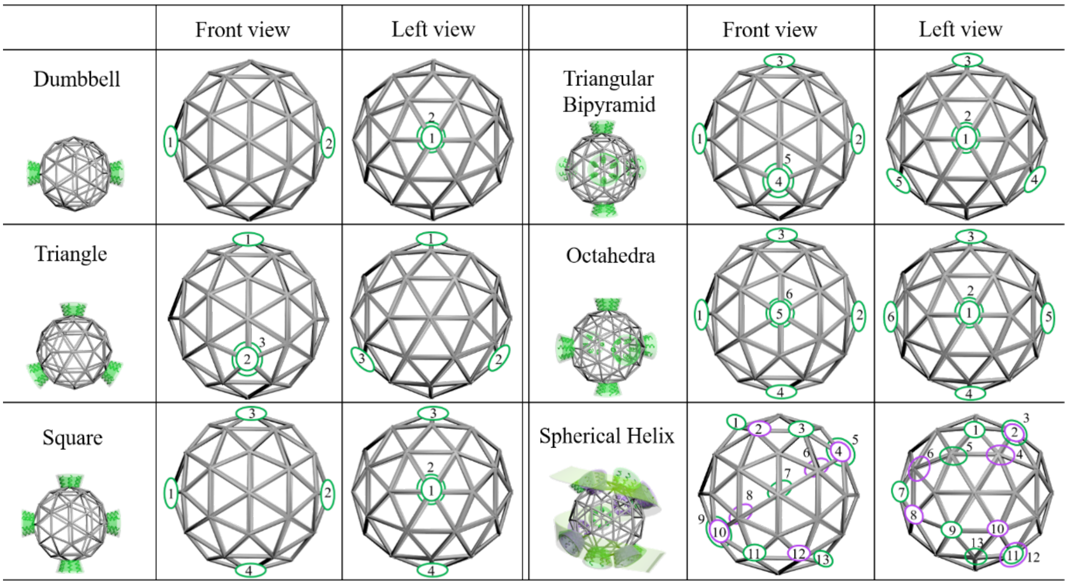

Supplementary Figure 1. Schematic illustration of different valence modes on the DNA meshframe. Encoding sites are numbered and shown in both front view and left view.

Theoretical interparticle distances of symmetric nanoclusters

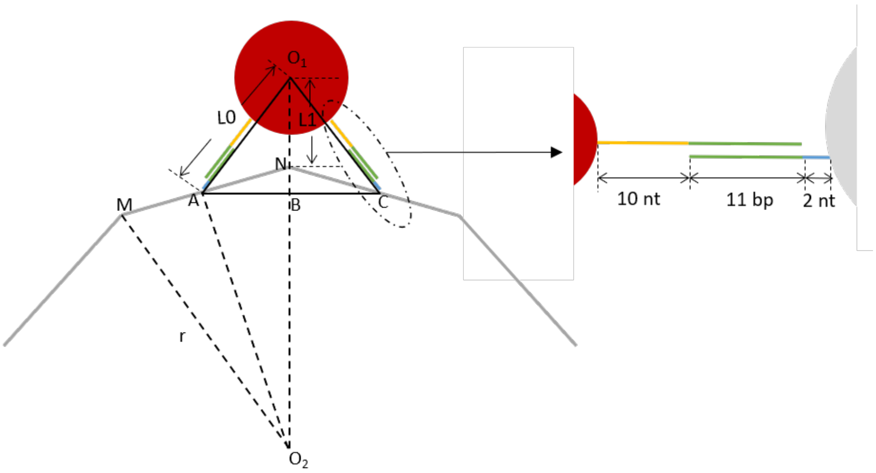

Supplementary Figure 2. Schematic illustration of AuNP attachment on the DNA meshframe. Red ball indicates 10 nm AuNP. Grey lines indicate DNA meshframe and the average edge length is 47 base pairs (bp). Single-stranded DNA attached on AuNP consists of 10-base inner spacer region (orange line) and 11-base outer recognition region (green line). Sticky ends protruded from the edge center of DNA wireframe consist of 2-base inner spacer region (blue line) and 11-base outer recognition region (green line).

Here we assume that double-stranded DNA is 3.5 nm/10.5 bp and single-stranded DNA is 0.3 nm/nucleotide.

The radius of the DNA meshframe

$$r = M\,O_2 = N\,O_2 = 25.1\,nm$$

and edge length (47 bp)

$$MN = 15.7\,nm$$

and

$$L_0 = 12.2\,nm$$

Therefore, we can get

$$L_1 = 7.3\,nm$$

and the theoretical interparticle distance,

$$d_1 = 2(r + L_1) = 64.8\,nm$$

$$d_2 = d_1 cos\frac{\pi}{6} = 56.1\,nm$$

$$d_3 = d_1 sin\frac{\pi}{4} = 45.8\,nm$$

Interparticle distances of nanoclusters fitted from SAXS

For a *N*-particle system,

$$I(q) = <|\sum_{j=1}^{N} f_j\, e^{i\cdot \boldsymbol{q}\cdot \boldsymbol{d}_j}|^2>_{orien.ave.}$$

$$= \sum_{j=1}^{N} |f_j|^2 + \sum_{\substack{j=1\\k>j}}^{N} 2 f_j f_k \frac{sin(qd_{jk})}{qd_{jk}} \tag{1}$$

For identical particle system,

$$f_j = f_k = f$$

Therefore,

$$I(q) = Nf^2 + \sum_{\substack{j=1\\k>j}}^{N} 2\ f^2 \cdot \frac{sin\ (qd_{jk})}{qd_{jk}} \tag{2}$$

Due to

$$S(q) = \frac{I(q)}{Nf^2}$$

Therefore,

$$S(q) = 1 + \frac{2}{N}\sum_{\substack{j=1\\k>j}}^{N} \frac{sin\ (qd_{jk})}{qd_{jk}} \tag{3}$$

For the symmetric nanoclusters, the geometries are shown below. (I) to (V) represent the geometry of dumbbell, triangular, square, TBP and octahedral cluster, respectively. The grey dot-dash circle in (I), (II) and (III) indicates the cross section of the DNA meshframe.

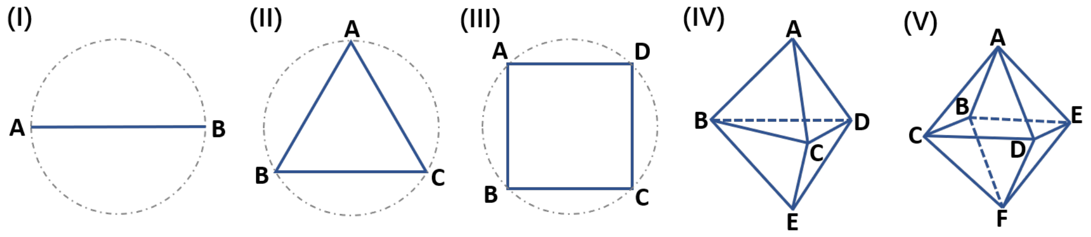

(I)	AB = d_1
(II)	AB=BC=AC= d_2
(III)	AC=BD= d_1
	AB=BC=CD=DA= d_3
(IV)	AE= d_1
	BC=CD=BD= d_2
	AB=AC=AD=EB=EC=ED= d_3
(V)	AF=BD=CE= d_1
	AB=AC=AD=AE=FB=FC=FD=FE=BC=CD=DE=BE= d_3

For dumbbell cluster, $N = 2, d_{jk} = d_1$

$$S(q)_{dumbbell} = 1 + \frac{sin\,(qd_1)}{qd_1} \tag{4}$$

For triangular cluster, $N = 3, d_{jk} = d_2$

$$S(q)_{tri} = 1 + \frac{2}{3} \cdot \frac{sin\,(qd_2)}{qd_2} \tag{5}$$

For square cluster, $N = 4, d_{jk} = d_1, d_3$

$$S(q)_{square} = 1 + \frac{1}{2} \cdot (2 \cdot \frac{\sin(qd_1)}{qd_1} + 4 \cdot \frac{\sin(qd_3)}{qd_3}) \tag{6}$$

For TBP cluster, $N = 5, d_{jk} = d_1, d_2, d_3$

$$S(q)_{TBP} = 1 + \frac{2}{5} \cdot (\frac{\sin(qd_1)}{qd_1} + 3 \cdot \frac{\sin(qd_2)}{qd_2} + 6 \cdot \frac{\sin(qd_3)}{qd_3}) \tag{7}$$

For octahedral cluster, $N = 6, d_{jk} = d_1, d_3$

$$S(q)_{OCT} = 1 + \frac{1}{3} \cdot (3 \cdot \frac{\sin(qd_1)}{qd_1} + 12 \cdot \frac{\sin(qd_3)}{qd_3}) \tag{8}$$

Supplementary Table 1. Interparticle distances of symmetric nanoclusters fitted from SAXS.

	Dumbbell	Triangle	Square	TBP	Octahedra
d_1 (nm)	68.4		68.3	67.9	67.6
d_2 (nm)		58.0		58.2	
d_3 (nm)			47.4	46.8	47.3

SAXS analysis and theoretical calculations of spherical helix clusters

For spherical helix cluster, $N = 13$, there are 78 correlation distances of which 29 are unique. The theoretical $S(q)$ is calculated using the pair distribution function (PDF). The contributions to theoretical $S(q)$ from 1st nearest-neighbors (NNs), 2nd NNs and longer-range correlations are highlighted in Figure S3b,c. One can see that the large number of unique correlation lengths provide a distinct, fine structure to theoretical $S(q)$ (black line in Supplementary Figure 3b); however, in a real system, with a certain degree of interparticle distance distribution, the $S(q)$'s fine structure smears out due to the rapid decay of higher-order harmonic components (associated with each of the present interparticle correlations, i.e. PDF).

The experimental $S(q)$ for the spherical helix cluster, as shown in Supplementary Figure 4, shows three peaks that are indexed to the primary harmonics of 1st NNs (q_2), 2nd NNs (q_1) and longer-range correlations (q_0). The first two peaks (q_0, q_1) match well with the theoretical $S(q)$ and corresponding primary harmonics. The third peak is shifted to the lower q (larger distances). That agrees with the tomographic measurements, which show that a correlation distance of 1st NNs in the synthesized helix clusters is 2.5 nm larger than in the design, possibly due to the steric repulsions between neighboring particles. The smearing of the higher-order peaks by interparticle distance distribution (polydispersity of the correlation lengths) is verified by averaging the calculated $S(q)$ using three independent sets of nanoparticle positions of the spherical helix clusters, as obtained by the cryo-TEM tomography. This calculated $S(q)$ clearly shows a quick diminishing of the higher-order peaks even in such a small population. The effect of polydisperse distances between nanoparticle is much less pronounced in other symmetric cluster systems (Figure 3 in the main text) since they only have a maximum of three unique correlation lengths and those lengths are significantly larger, which practically eliminates steric repulsions.

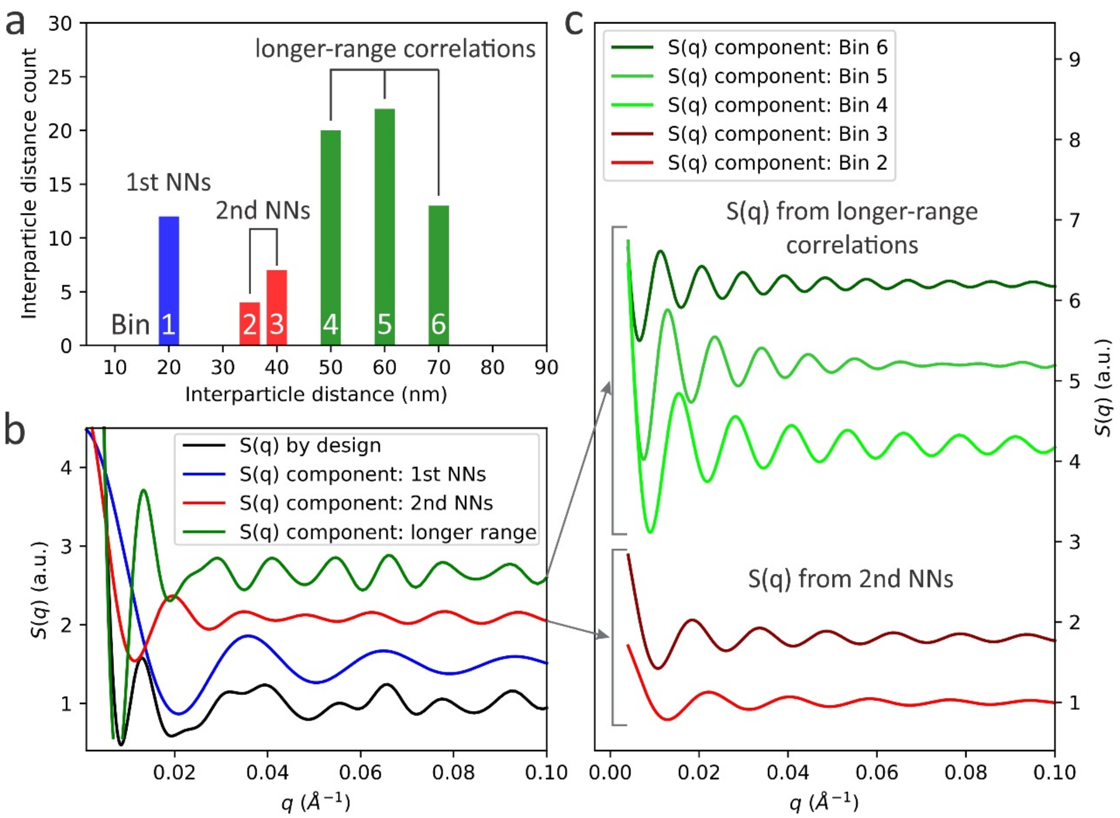

Supplementary Figure 3. Theoretical calculation of structure factor *S(q)* of the designed spherical helix cluster. a, Histogram plot (pair distribution function) of the interparticle distances in the spherical helix cluster. The spherical helix cluster has 78 correlation distances of which 29 are unique. These distances can be grouped into six bins with a constant bin width of 5 nm, and further into three types of correlations—1st nearest-neighbors (NNs), 2nd NNs and longer-range correlations. b, Theoretical *S(q)* of the designed spherical helix cluster and the *S(q)* components from the different types of correlations. c, *S(q)* components as corresponding to bins in 3a for the 2nd NNs and the longer-range correlations.

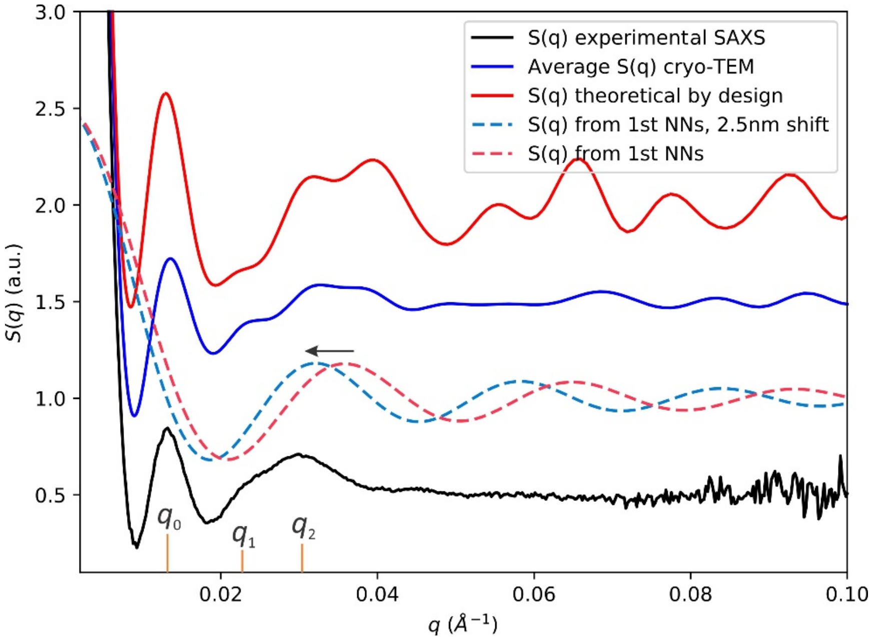

Supplementary Figure 4. Structure factors *S(q)* of the spherical helix clusters, as obtained from SAXS experiment and from computation. Computed structure factors *S(q)* for the designed spherical helix cluster (red), average *S(q)* calculated from nanoparticle positions in cryo-TEM tomography reconstruction (blue), *S(q)* derived from experiment SAXS (black), and *S(q)* components calculated from 1st NNs for designed cluster (red dash) and from 1st NNs that have 2.5 nm larger interparticle distances than the designed ones (blue dash). The 2.5 nm shift is based on the tomographic measurements.

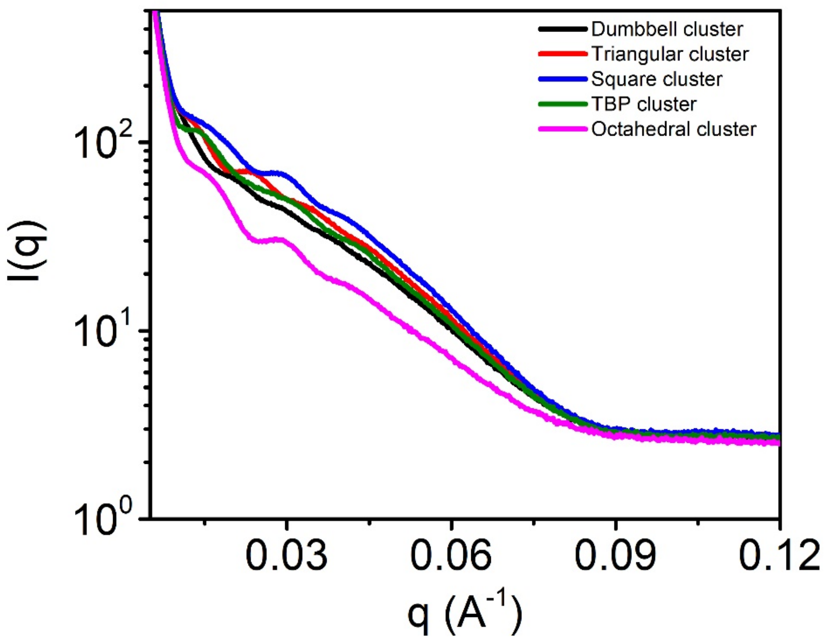

Supplementary Figure 5. 1D scattering intensity of symmetric nanoparticle clusters.

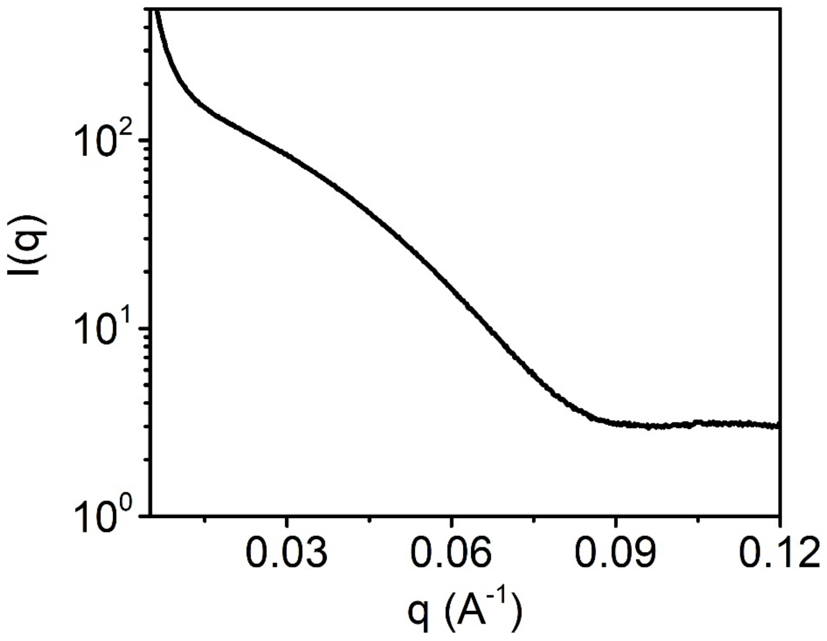

Supplementary Figure 6. 1D scattering intensity of free gold nanoparticles.

Supplementary Figures

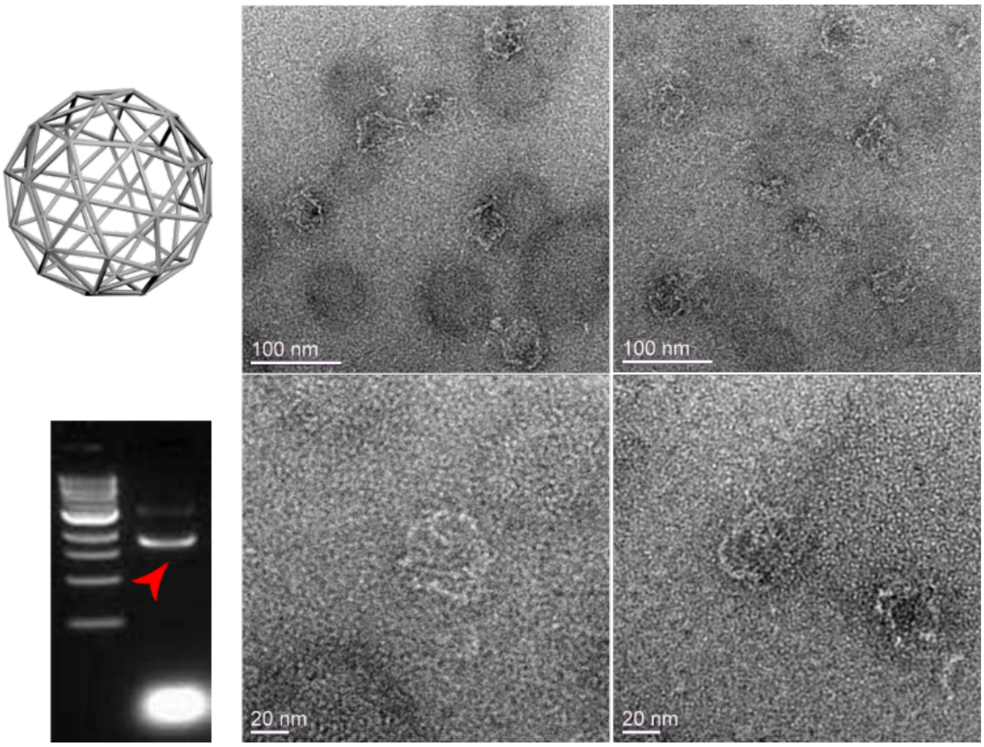

Supplementary Figure 7. Agarose gel electrophoresis and negative-stained TEM images of DNA meshframe. Red arrow indicates the product band on the agarose gel. The band at the bottom of the gel is excess single-stranded DNA. Bands in the left lane is 1kb DNA ladder.

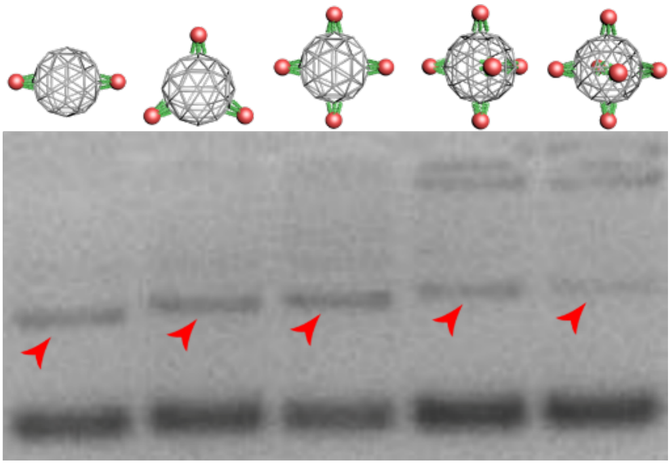

Supplementary Figure 8. Agarose gel electrophoresis of 10 nm AuNP clusters under white light. Lanes from left to right correspond to dumbbell, triangular, square, TBP and octahedral nanoclusters, respectively. Red arrows indicate product bands. Bands at the bottom of the gel are excess 10 nm AuNPs.

We estimated overall cluster yield by analyzing the band intensity on agarose gel under ambient light taking into account misfolded DNA origami and yield from TEM data. The estimated yields are: $yield_{dumbbell} = 77\%$, $yield_{triangle} = 44\%$, $yield_{square} = 49\%$, $yield_{TBP} = 39\%$, $yield_{octahedra} = 20\%$.

Typically, we use 0.4 picomoles of scaffold plasmid per synthesis. In a typical preparation we make about 10^{11} clusters.

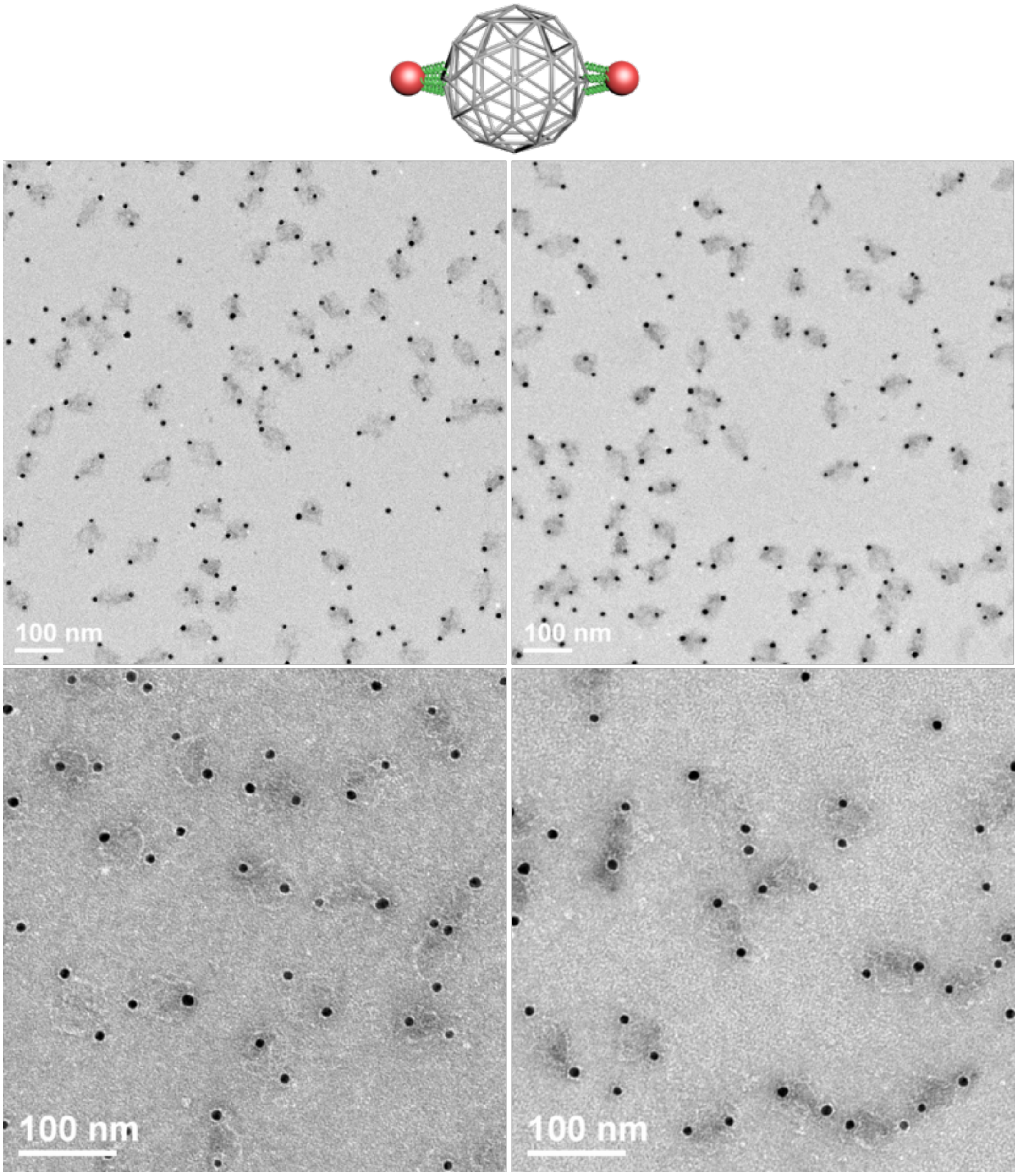

Supplementary Figure 9. Additional negative-stained TEM images of dumbbell clusters.

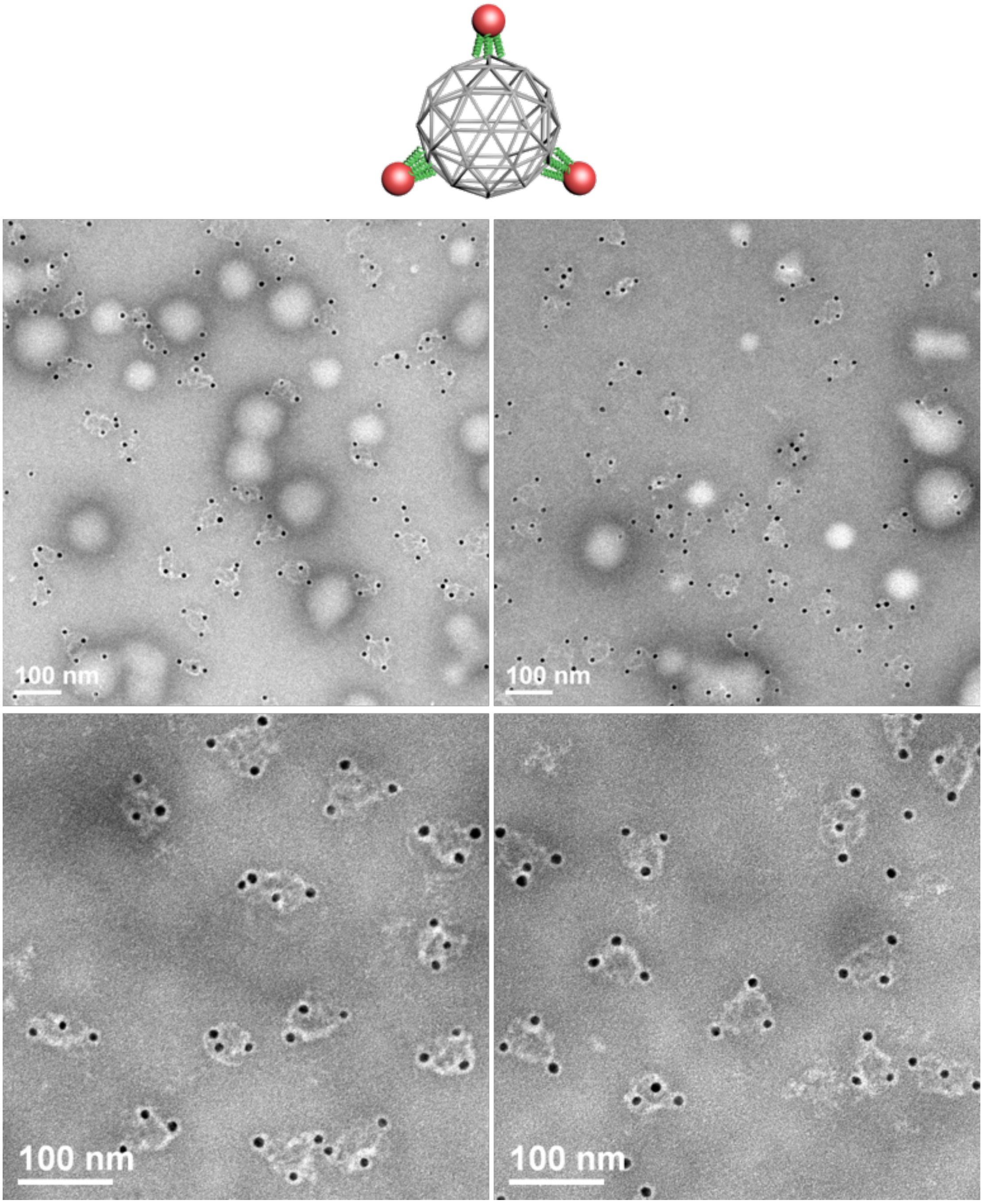

Supplementary Figure 10. Additional negative-stained TEM images of triangular clusters.

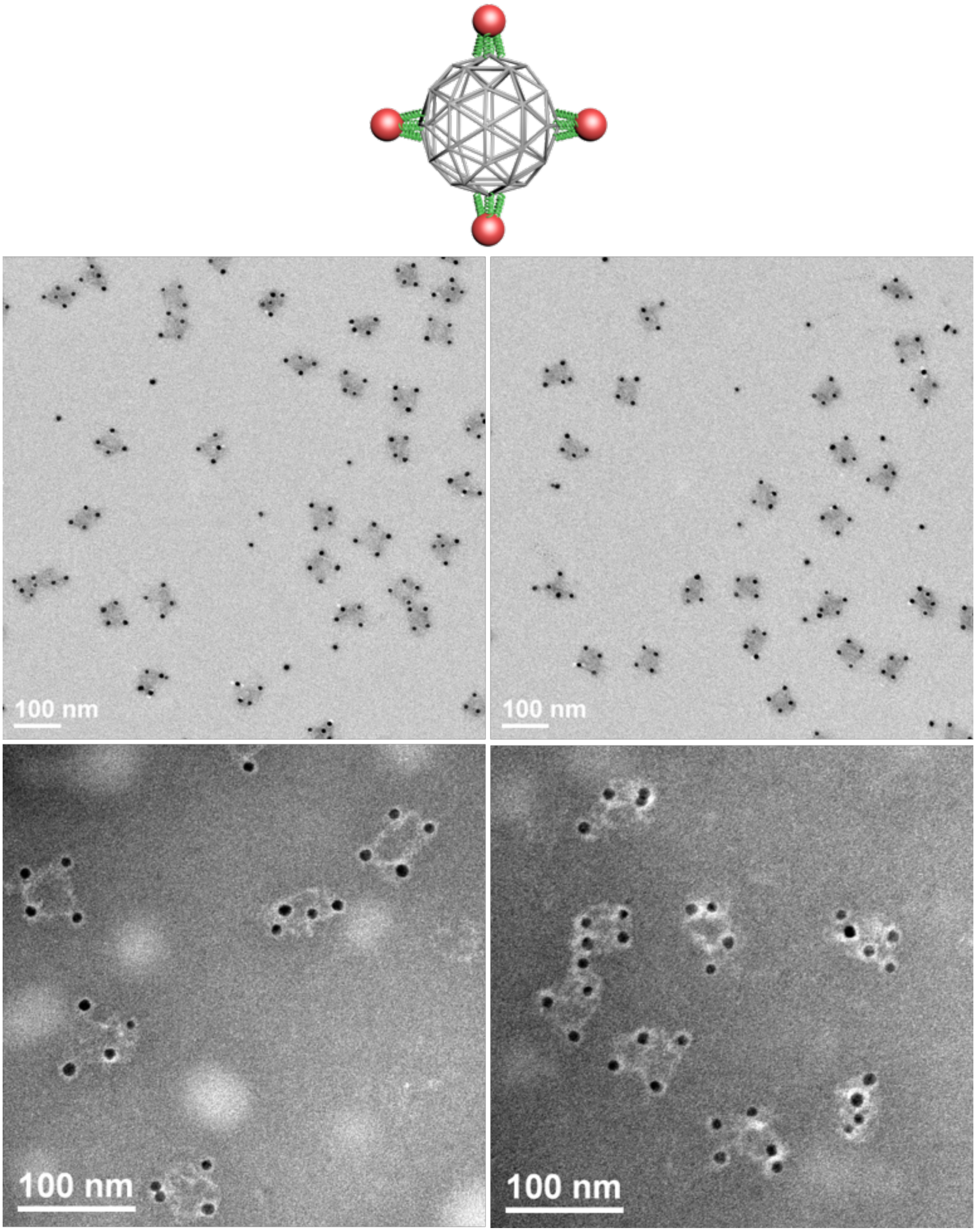

Supplementary Figure 11. Additional negative-stained TEM images of square clusters.

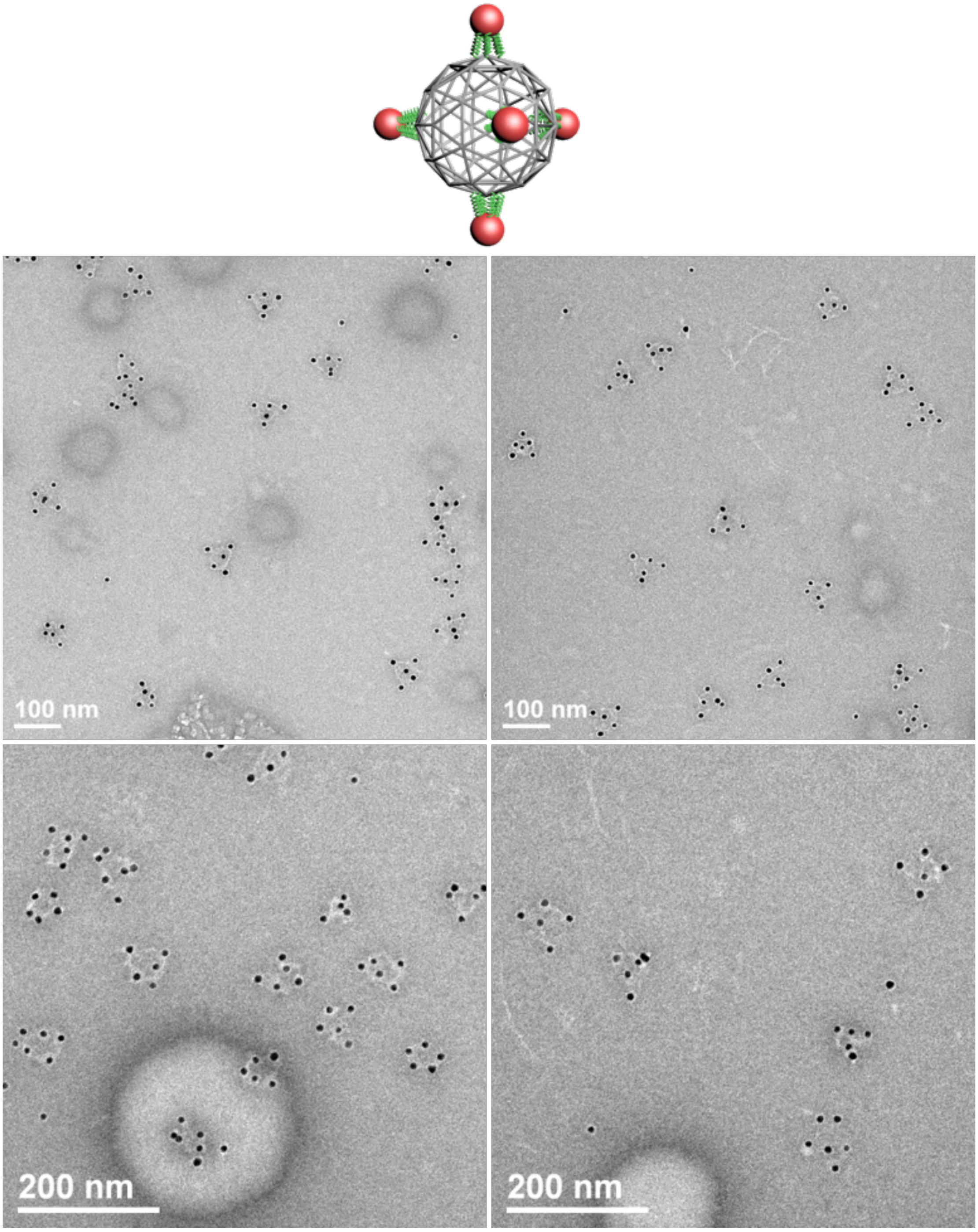

Supplementary Figure 12. Additional negative-stained TEM images of TBP clusters.

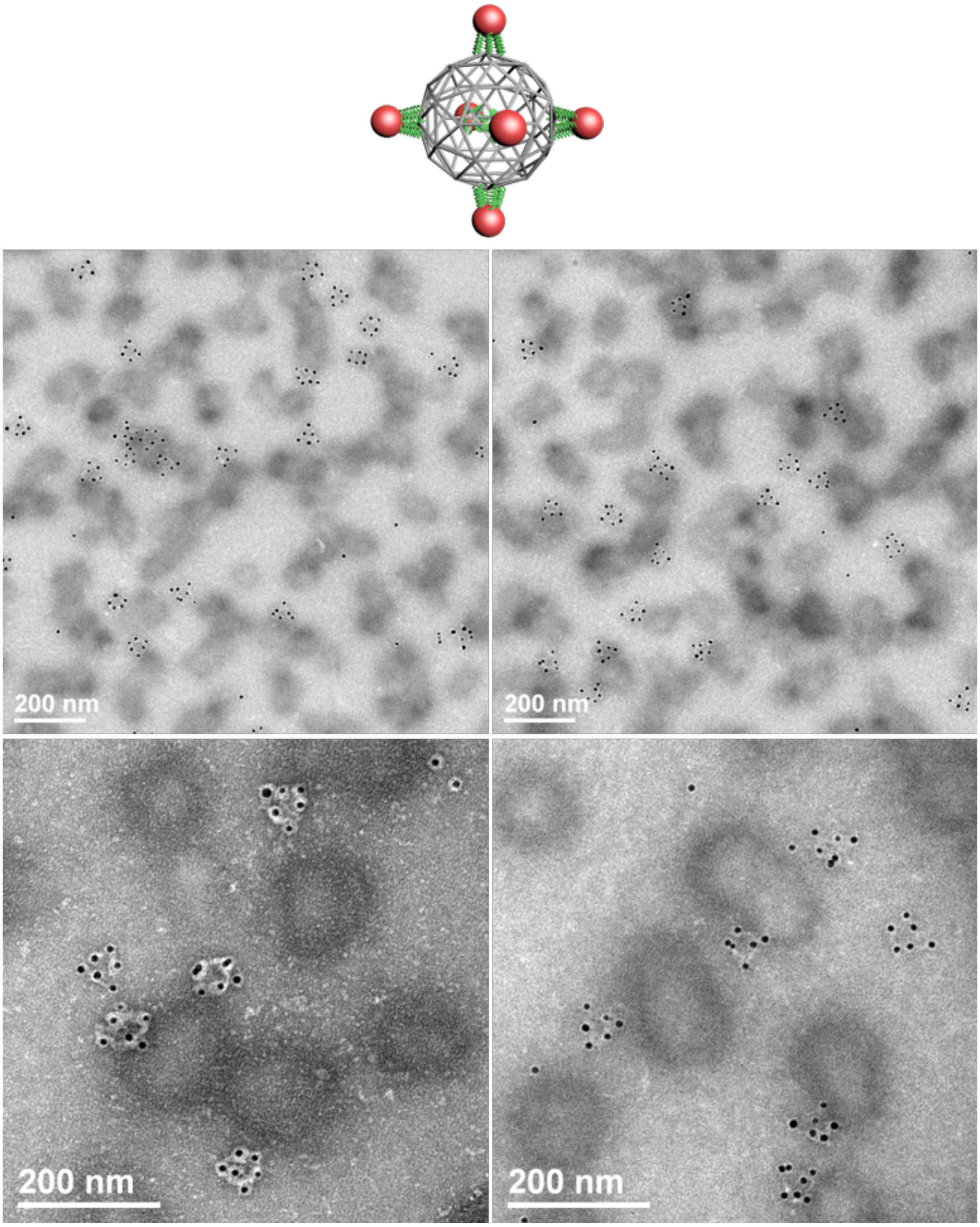

Supplementary Figure 13. Additional negative-stained TEM images of octahedral clusters.

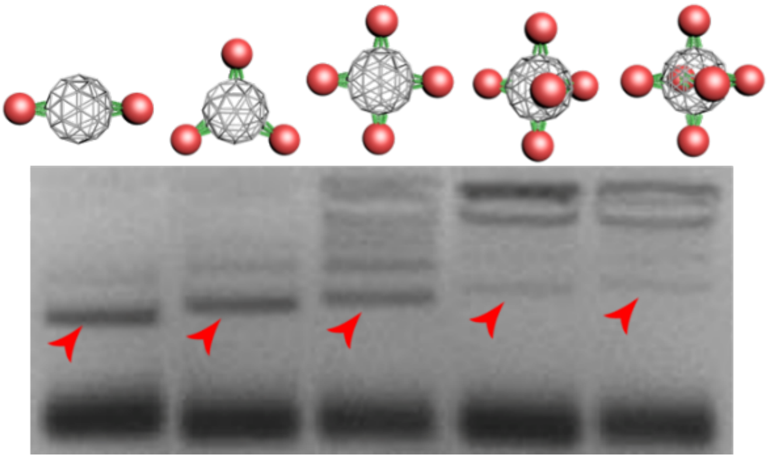

Supplementary Figure 14. Agarose gel electrophoresis of 20 nm AuNP clusters under white light. Lanes from left to right correspond to dumbbell, triangular, square, TBP and octahedral clusters, respectively. Red arrows indicate product bands. Bands at the bottom of the gel are excess 20 nm AuNPs.

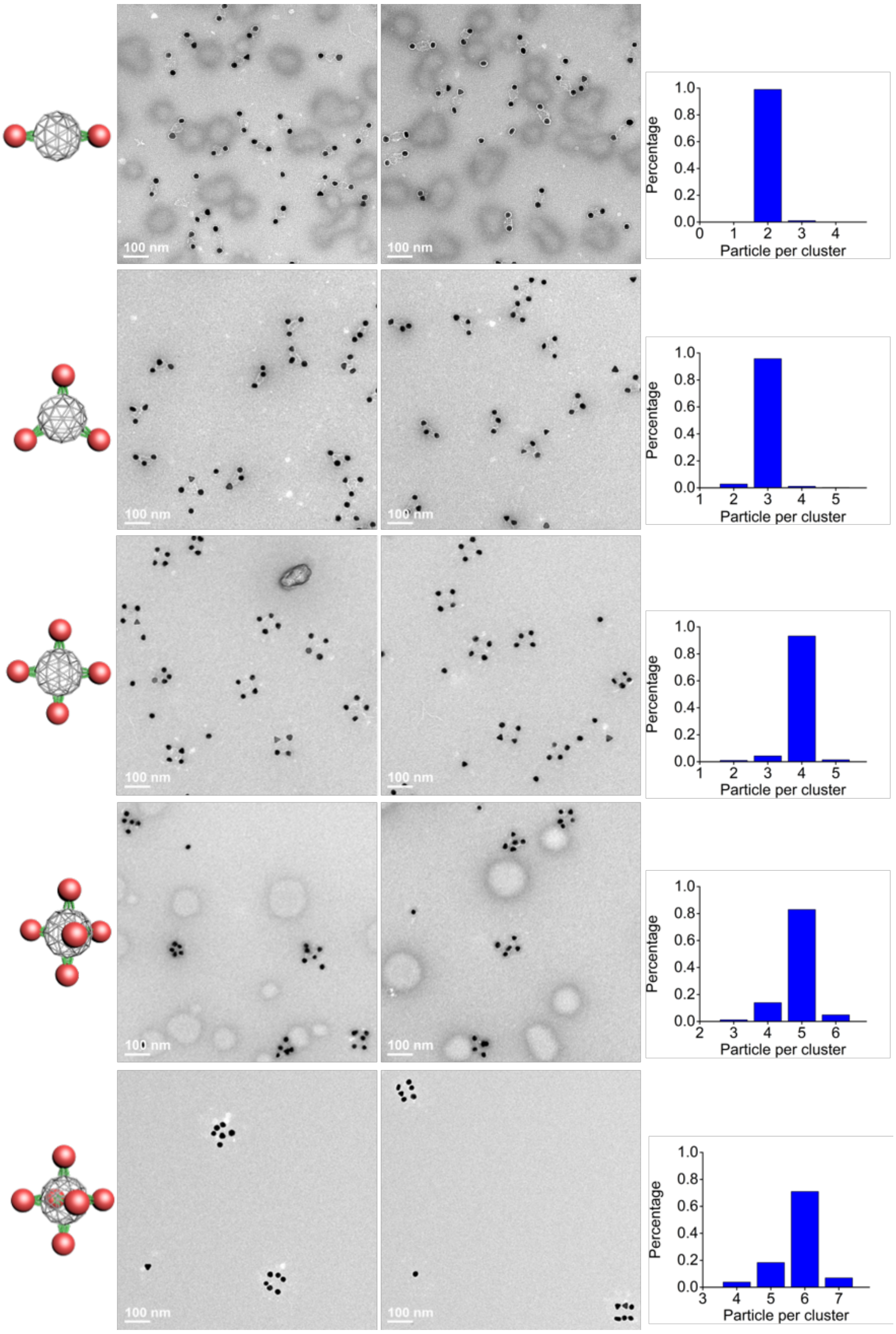

Supplementary Figure 15. Representative TEM images and population histograms of 20 nm AuNP clusters.

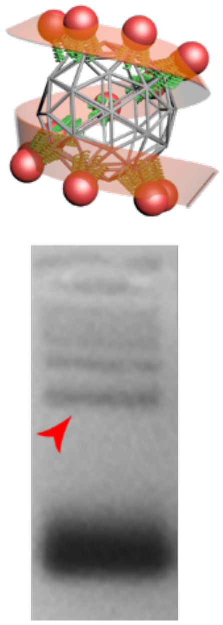

Supplementary Figure 16. Agarose gel electrophoresis of spherical helix cluster under white light. Red arrow indicates the product band. Band at the bottom of the gel is excess 10 nm AuNPs.

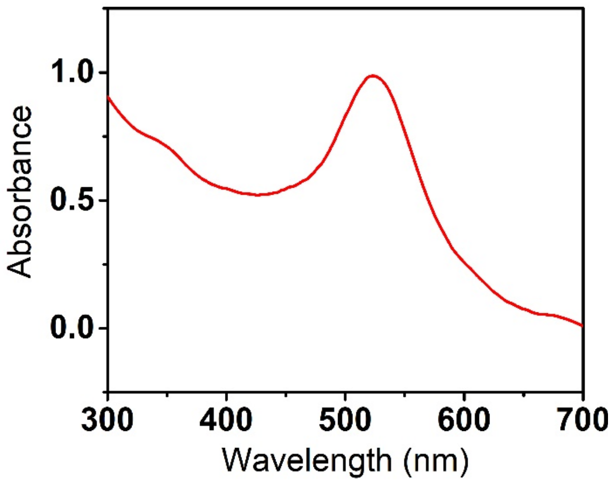

Supplementary Figure 17. Normalized UV-vis absorbance of purified spherical helix clusters. The absorption peak is at 520 nm.

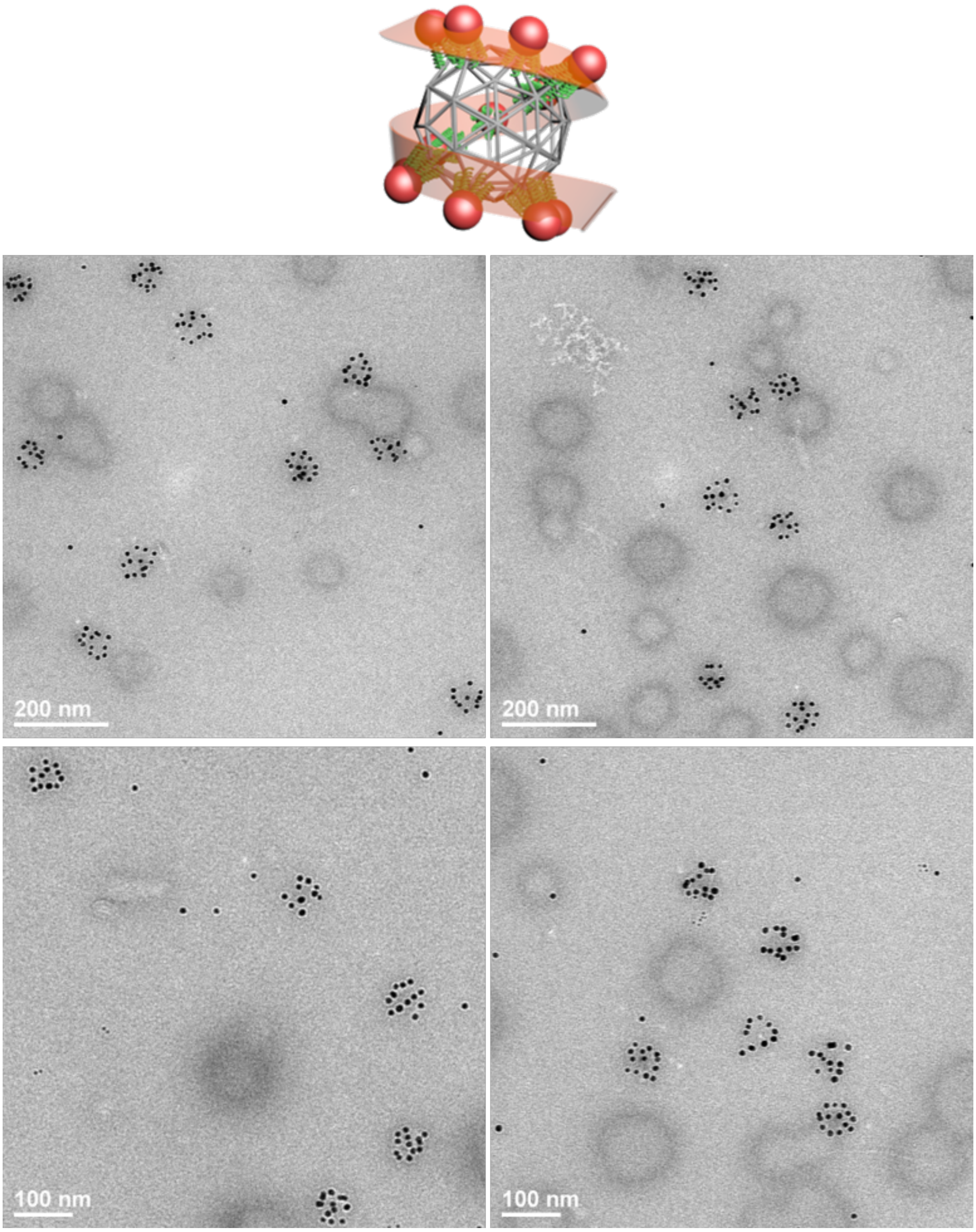

Supplementary Figure 18. Additional negative-stained TEM images of spherical helix clusters.

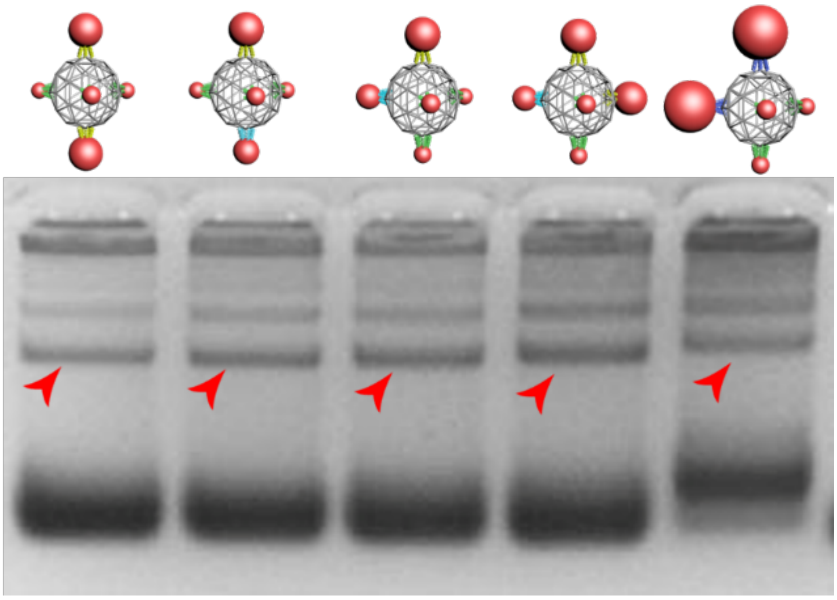

Supplementary Figure 19. Agarose gel electrophoresis of multi-type clusters under white light. Lanes from left to right correspond to $P_1(345)P_3(12)$, $P_1(345)P_2(2)P_3(1)$, $P_1(245)P_2(3)P_3(1)$, $P_1(24)P_2(3)P_3(15)$ and $P_1(245)P_4(13)$, respectively. Red arrows indicate product bands. Bands at the bottom of the gel are excess AuNPs.

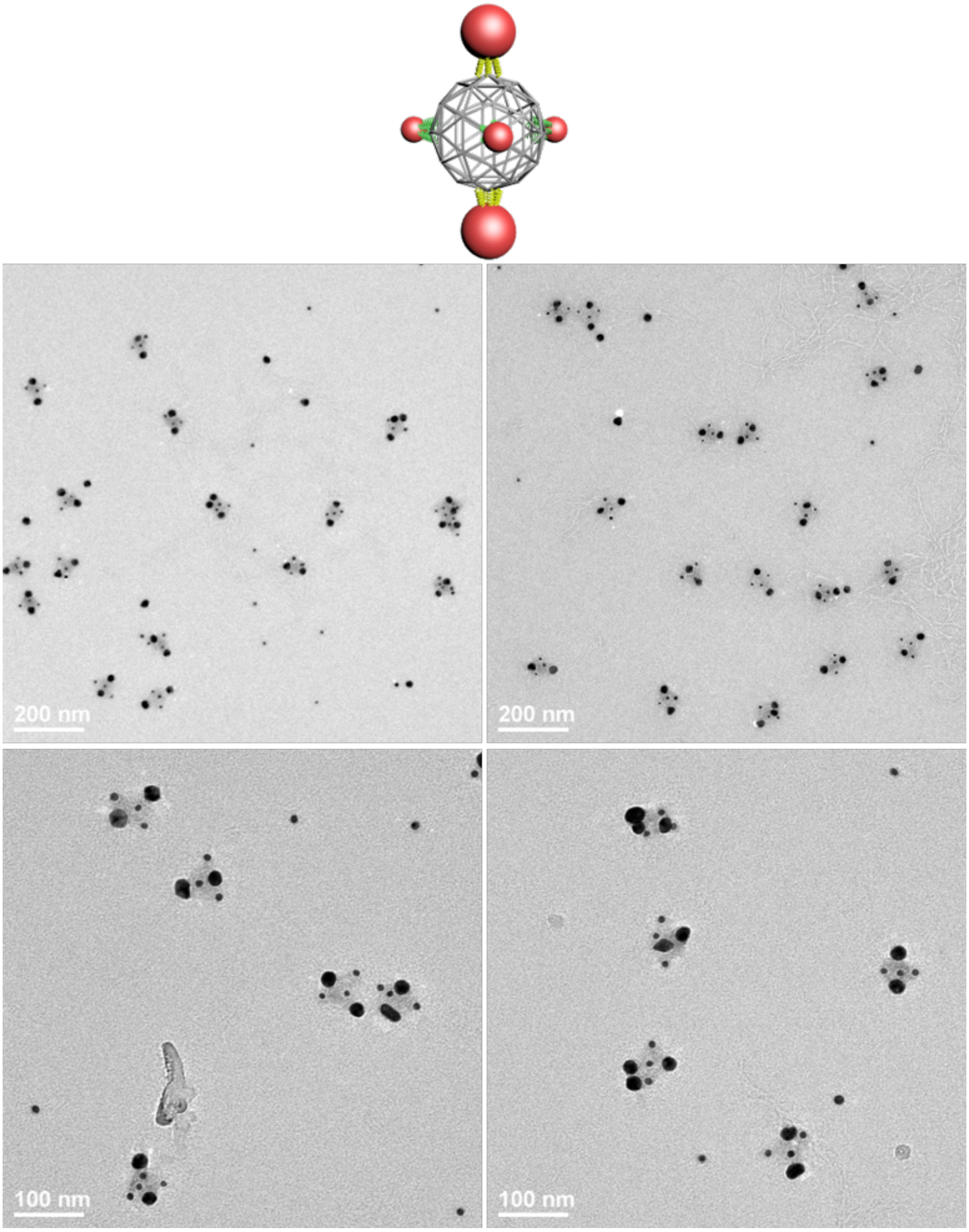

Supplementary Figure 20. Additional negative-stained TEM images of $P_1(345)P_3(12)$.

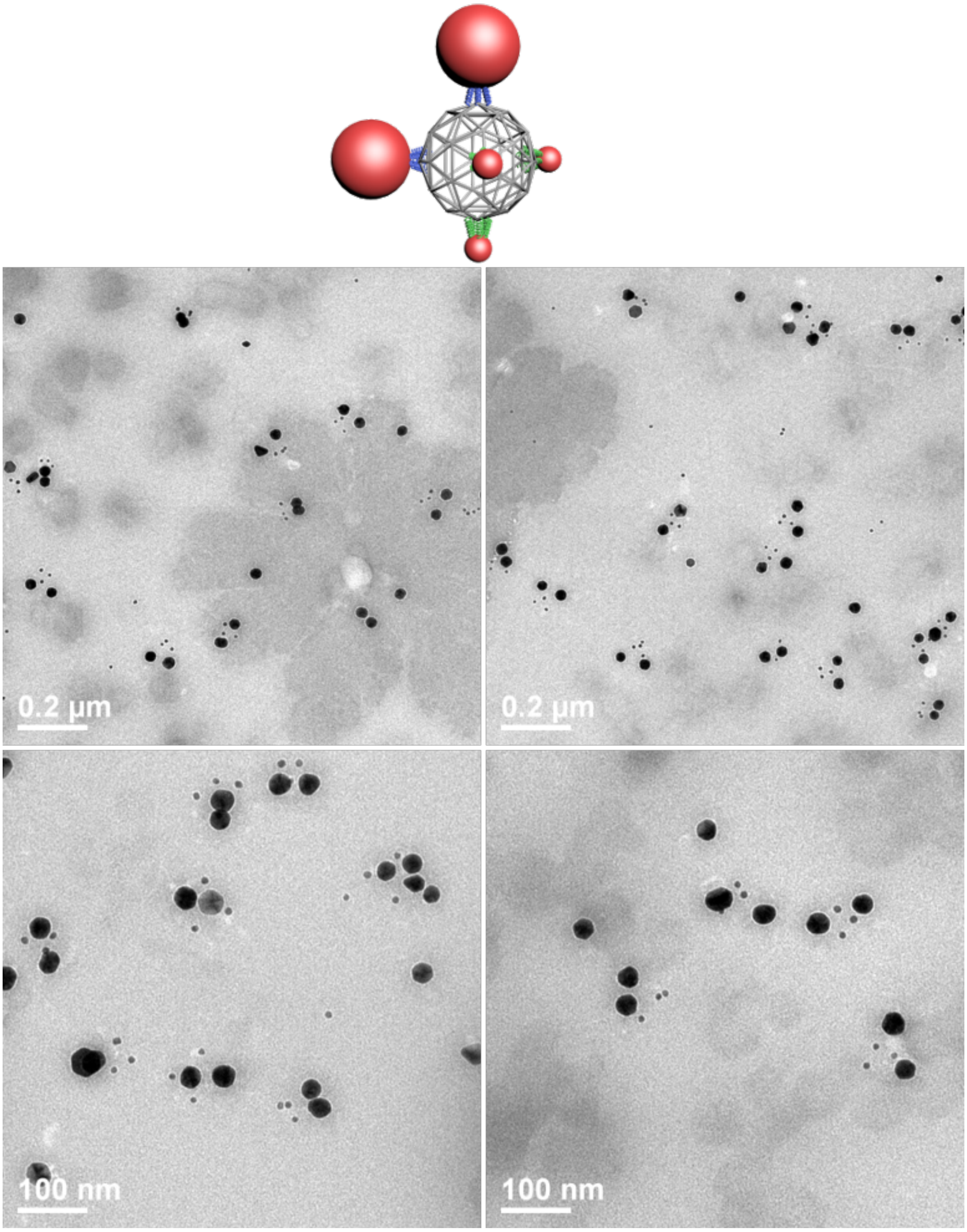

Supplementary Figure 21. Additional negative-stained TEM images of $P_1(245)P_4(13)$.

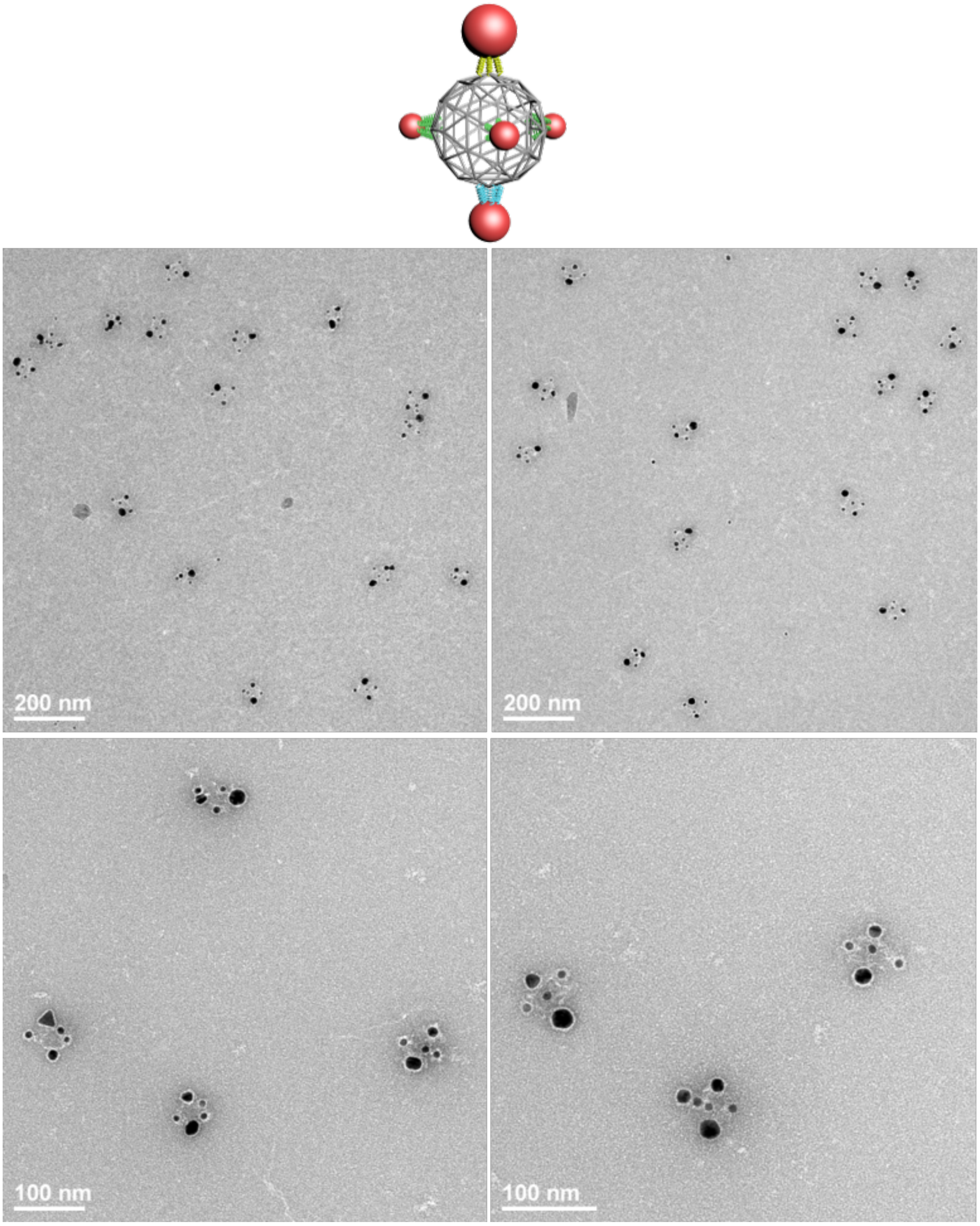

Supplementary Figure 22. Additional negative-stained TEM images of $P_1(345)P_2(2)P_3(1)$.

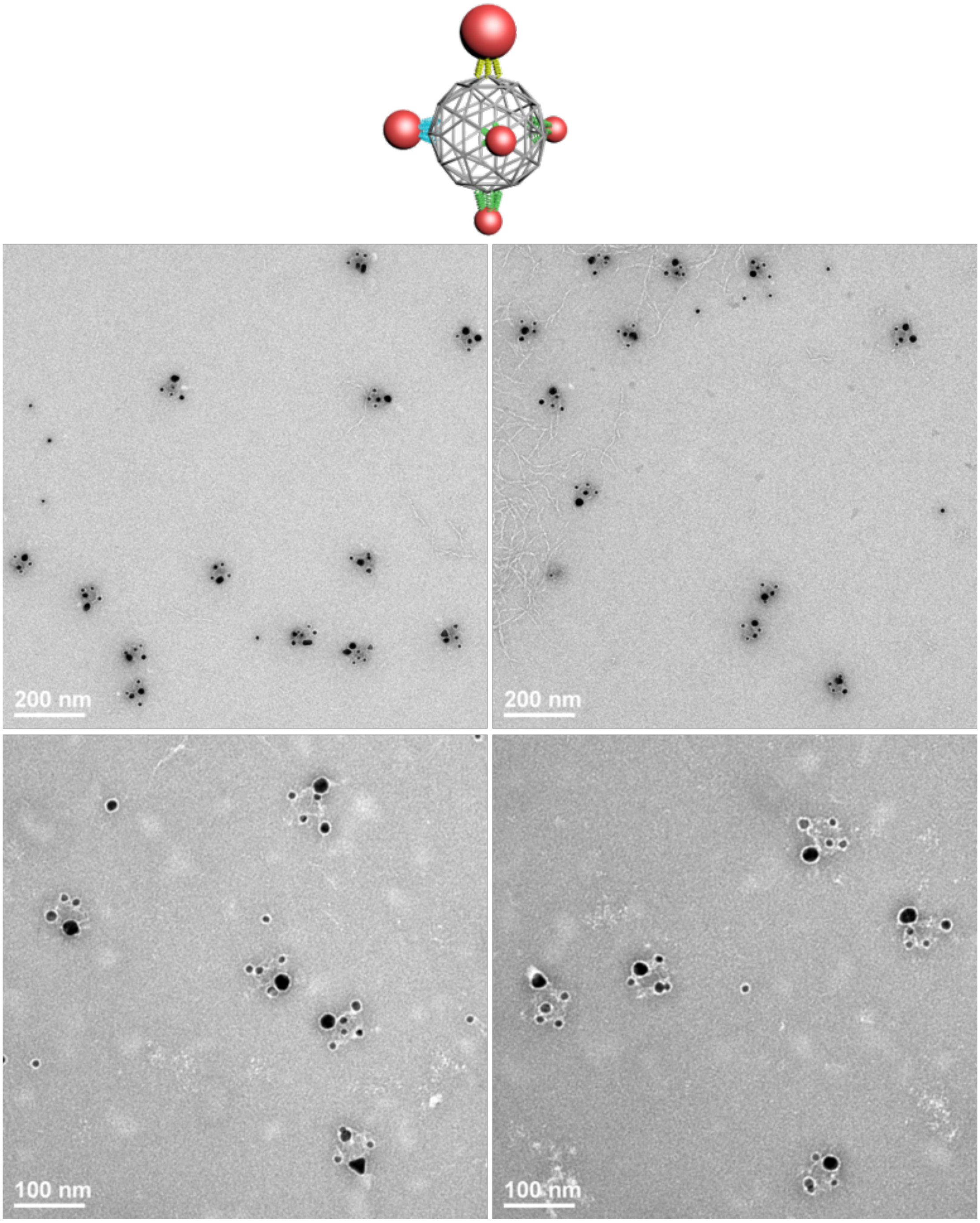

Supplementary Figure 23. Additional negative-stained TEM images of $P_1(245)P_2(3)P_3(1)$.

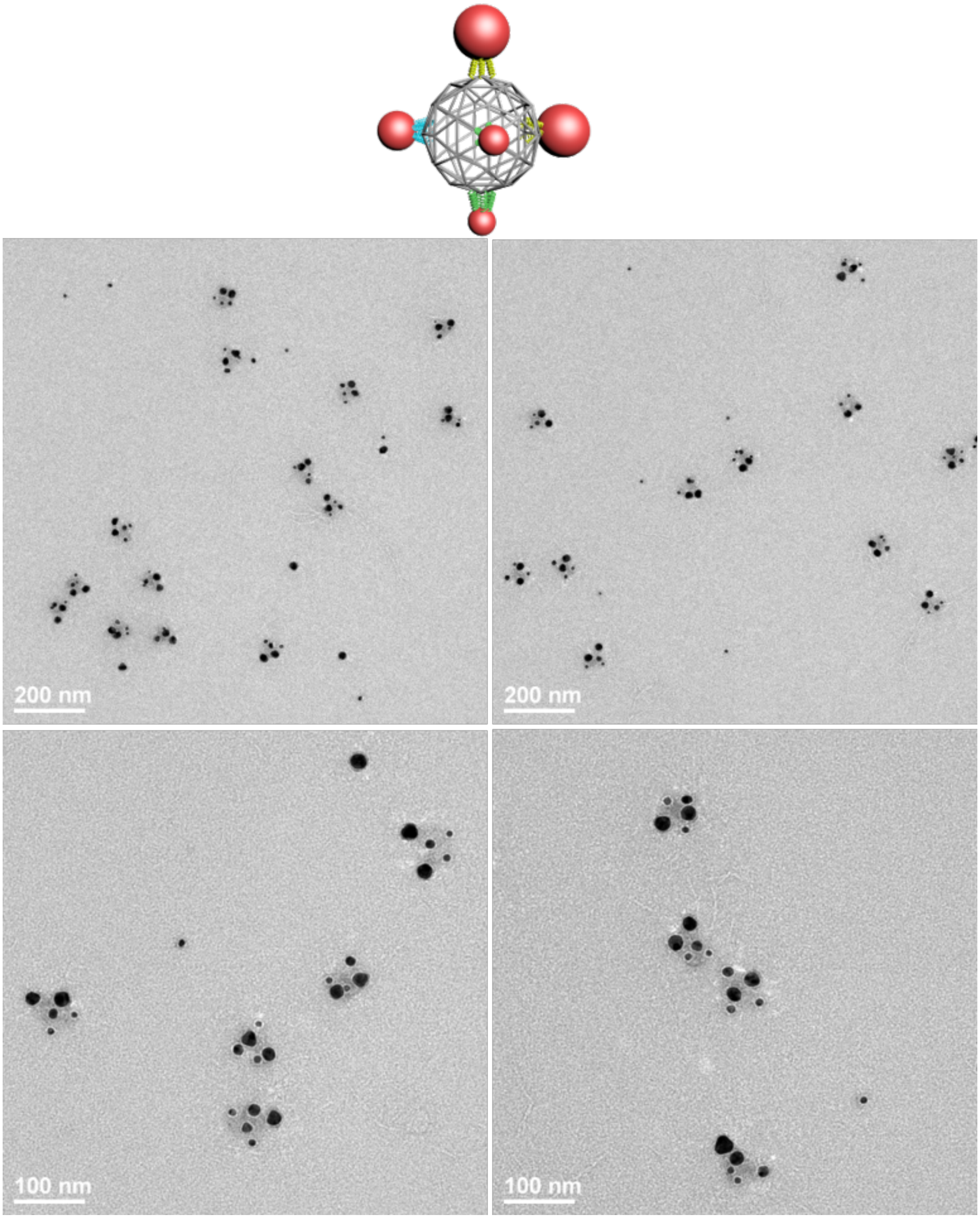

Supplementary Figure 24. Additional negative-stained TEM images of $P_1(24)P_2(3)P_3(15)$.

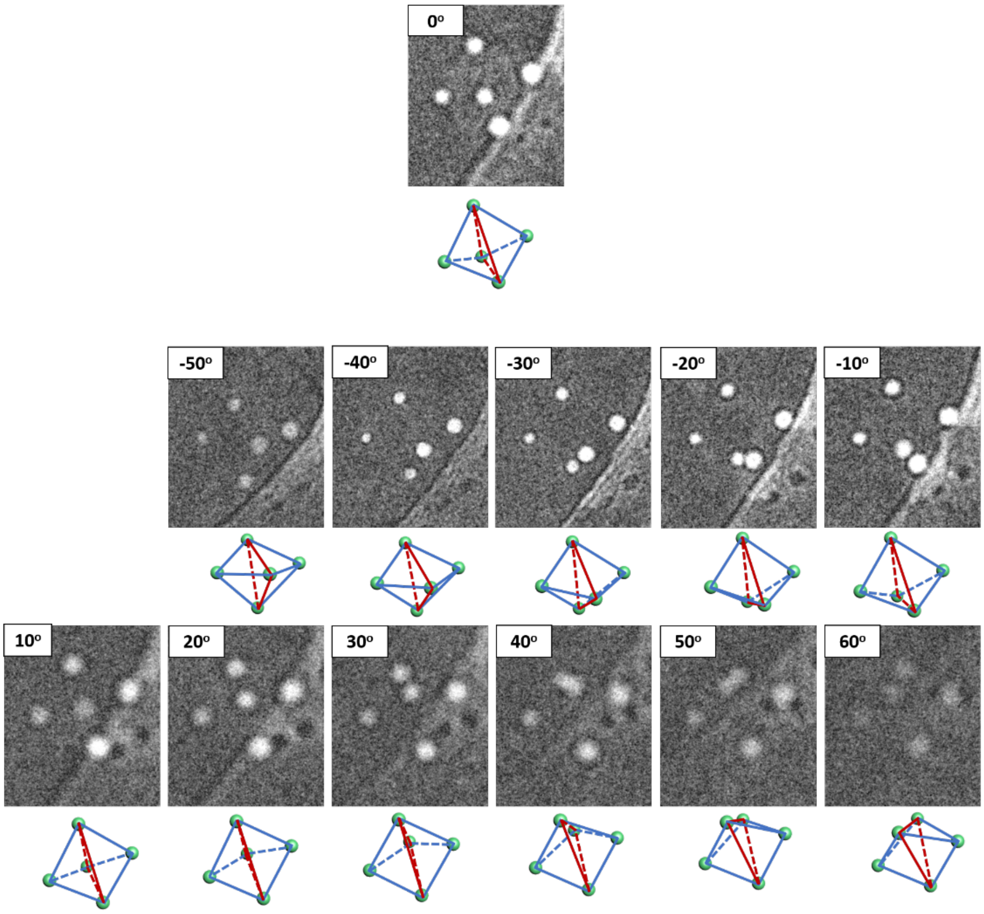

Supplementary Figure 25. Cryo-TEM images of TBP cluster tiled from -50° to 60° and corresponding reconstructed 3D structures. Red lines (solid and dashed) were drawn to connect three particles on the triangular plane. Dashed lines (red and blue) were drawn to give a clear 3D visual guidance. Cryo-TEM images are contrast flipped to facilitate the reconstruction process.

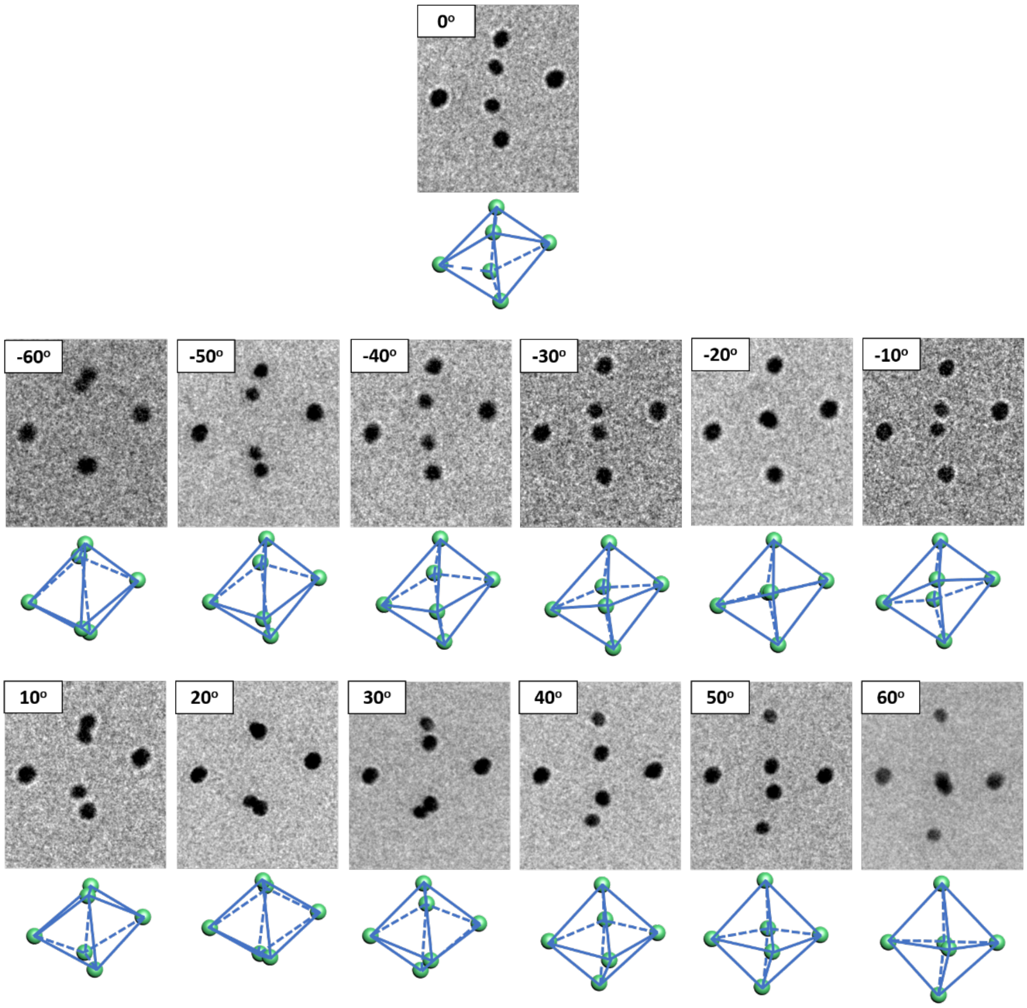

Supplementary Figure 26. Cryo-TEM images of octahedral cluster tilted from -60° to 60° and corresponding reconstructed 3D structures. Lines connecting NPs were drawn to give a clear 3D visual guidance.

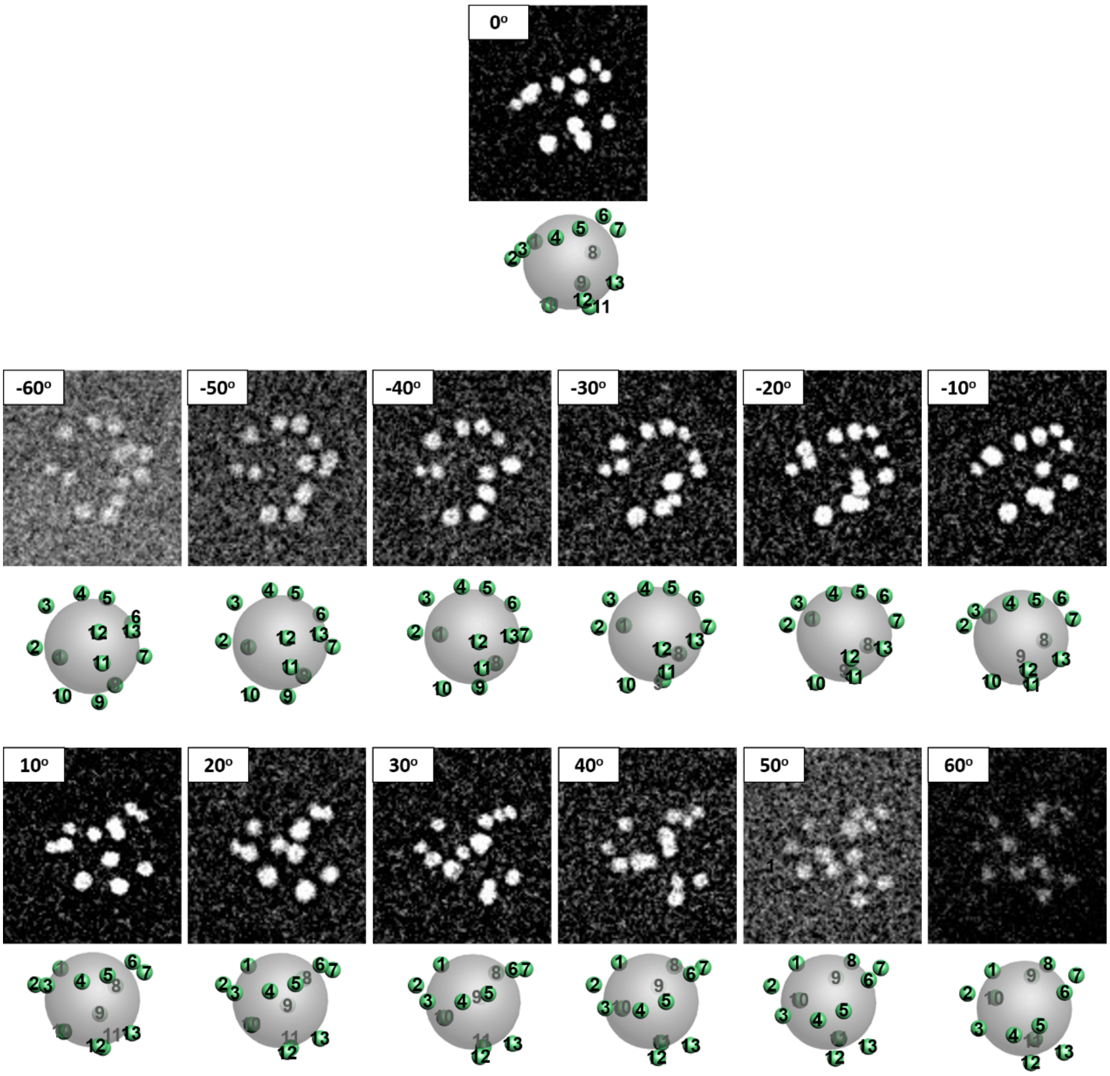

Supplementary Figure 27. Cryo-TEM images of spherical helix cluster tiled from -60º to 60º with corresponding reconstructed 3D structures. The grey sphere is added in the center to provide a visual guidance. Because AuNPs are attached on the sphere surface, the central coordinate and diameter of the sphere can be fitted by limited-memory BFGS method (L-BFGS-B) using the reconstructed coordinates of 13 NPs. Cryo-TEM images are contrast flipped to facilitate the reconstruction process.

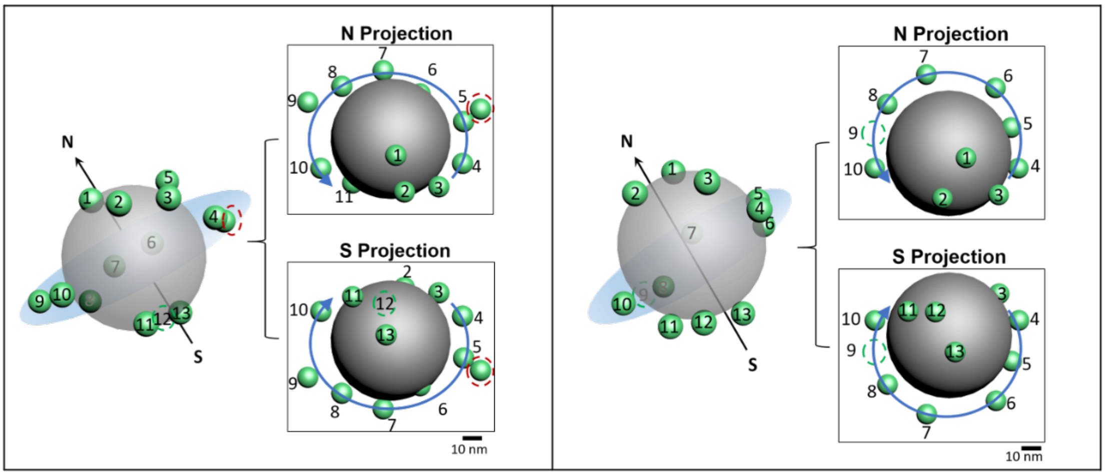

Supplementary Figure 28. Two independently reconstructed spherical helix clusters by cryo-TEM and tomography. Red dashed circle refers to mis-located nanoparticles and green dashed circle refers to absent nanoparticles.

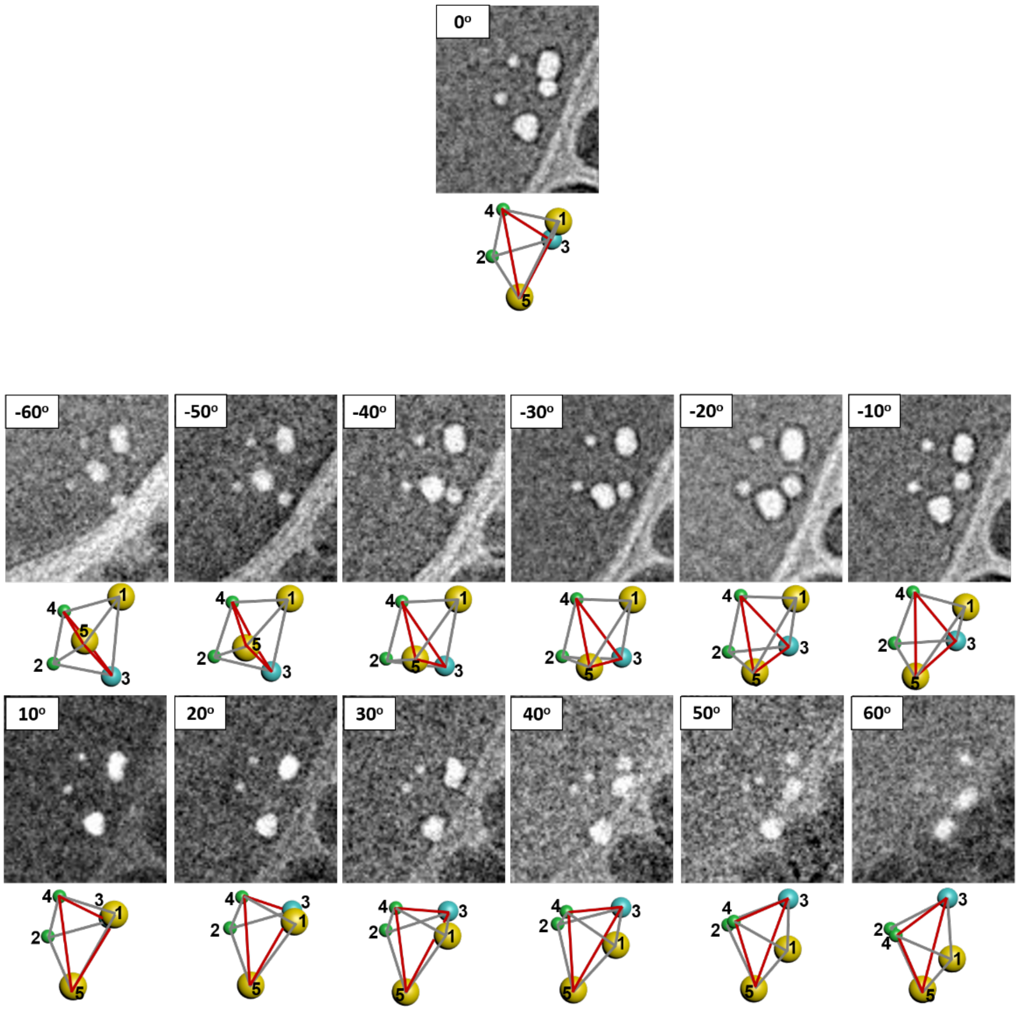

Supplementary Figure 29. Cryo-TEM images of $P_1(24)P_2(3)P_3(15)$ tiled from -60° to 60° with corresponding reconstructed 3D structures. Red lines were drawn to connect three particles on the triangular plane. Cryo-TEM images are contrast flipped to facilitate the reconstruction process.

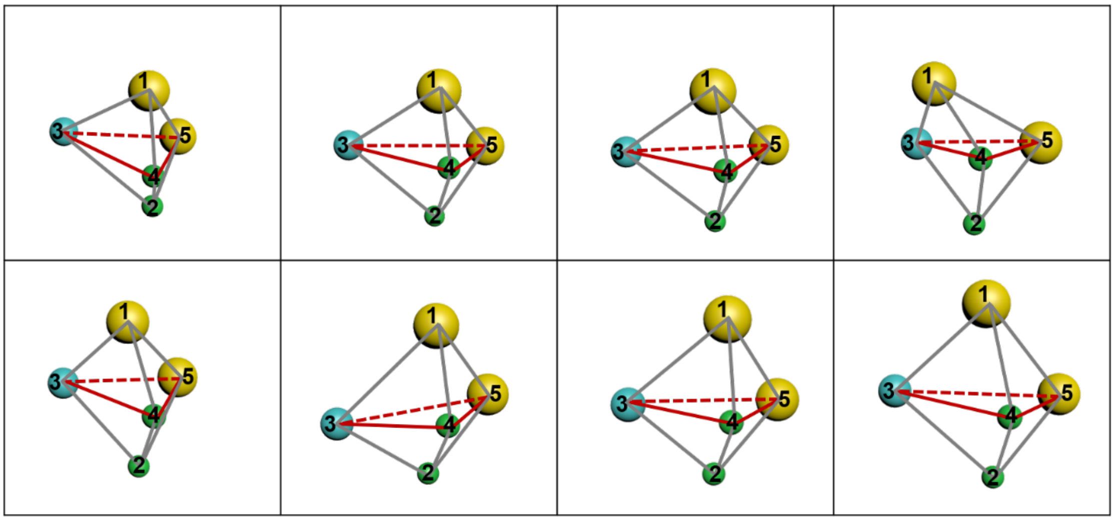

Supplementary Figure 30. Eight independently reconstructed $P_1(24)P_2(3)P_3(15)$ by cryo-EM tomography.

Supplementary Tables

Supplementary Table 2. Staple strands for sphere-like DNA meshframe.

Staple Name	Sequence
1	CCACCCTCAGAGCCACCACCCTCATAGCTATCTTACCGAAGCCCT
2	TTTTAAGAAAAGTAAGCAGATATAATCAAAATCACCGGAACCAG
3	AGCCACCACCGGAACCGCCTCCCACTATATGTAAATGCTGATGCAAATCC
4	AATCGCAAGACAAAGAACGCGAGAAATCGGCATTTTCGGTCATAGCCCCC
5	TTATTAGCGTTTGCCATCTTTTCATGTTAGCAAACGTAGAAAAT
6	ACATACATAAAGGTGGCAACACGACAGAATCAAGTTTGCCTTTAG
7	CGTCAGACTGTAGCGCGTTTTCAATCATATGCGTTATACAAATTCTTACC
8	AGTATAAAGCCAACGCTCAACAGTAGGGAAACGTCACCAATGAAACCATC
9	GATAGCAGCACCGTAATCAGTAGAAAGGTGAATTATCACCGTCACCG
10	ACTTGAGCCATTTGGGAATTAGATTTCATCGTAGGAATCATTAC
11	CGCGCCCAATAGCAAGCAAATCAACCGATTGAGGGAGGGAAGGTA
12	AATATTGACGGAAATTATTCATTTATAAAAGAAACGCAAAGACACCA
13	CGGAATAAGTTTATTTTGTCACAGGAATACCCAAAAGAACTGGCATG
14	ATTAAGACTCCTTATTACGCAGTAGCCGAACAAAGTTACCAGAAGGA
15	AACCGAGGAAACGCAATAATAACAATTGAGTTAAGCCCAATAATAAG
16	AGCAAGAAACAATGAAATAGCAATACCGTTCCAGTAAGCGTCATACATGG
17	CTTTTGATGATACAGGAGTGTACTGGAAGTCAGAGGGTAATTGAGCGCTA
18	ATATCAGAGAGATAACCCACAAGCAAAAATGAAAATAGCAGCCTTTA
19	CAGAGAGAATAACATAAAAACAGATATTATTTATCCCAATCCAAATA
20	AGAAACGATTTTTTGTTTAACGTATCAATAGAAAATTCATATGGTTTACC
21	AGCGCCAAAGACAAAAGGGCGACATTCGAGCGTCTTTCCAGAGCCTAATT
22	TGCCAGTTACAAAATAAACAGCCAGGTTTAGTACCGCCACCCTC
23	AGAACCGCCACCCTCAGAACCTATTTTGCACCCAGCTACAATTTT
24	ATCCTGAATCTTACCAACGCTAACAGATATAGAAGGCTTATCCGGTA
25	TTCTAAGAACGCGAGGCGTTTTAATTAAACCAAGTACCGCACTCATC
26	GAGAACAAGCAAGCCGTTTTTATAACCAATCAATAATCGGCTGTCTT
27	TCCTTATCATTCCAAGAACGGGTATAGTTGCGCCGACAATGACAACAACC
28	ATCGCCCACGCATAACCGATATATTCCCTGAACAAGAAAAATAATATCCC
29	ATCCTAATTTACGAGCATGTAGAGCCAGCAAAATCACCAGTAGC
30	ACCATTACCATTAGCAAGGCCATGTTCAGCTAATGCAGAACGCGC
31	CTGTTTATCAACAATAGATAAGTCCATTAAACGGGTAAAATACGTAATGC

32 CACTACGAAGGCACCAACCTAAAACGCCGACAAAAGGTAAAGTAATTCTG

33 TCCAGACGACGACAATAAACAACGGCTTAATTGAGAATCGCCATATT

34 TAACAACGCCAACATGTAATTTATAAGAATAAACACCGGAATCATAA

35 TTACTAGAAAAAGCCTGTTTAGTACTTTTTCAAATATATTTTAGTTA

36 ATTTCATCTTCTGACCTAAATTTGTCTGAGAGACTACCTTTTTAACC

37 TCCGGCTTAGGTTGGGTTATATATTAATTTTCCCTTAGAATCCTTGA

38 AAACATAGCGATAGCTTAGATTACATTTAACAATTTCATTTGAATTA

39 CCTTTTTTAATGGAAACAGTACAAGCAAAAGAAGATGATGAAACAAA

40 CATCAAGAAAACAAAATTAATTAAAAGAATAGCCCGAGATAGGG

41 TTGAGTGTTGTTCCAGTTTGGCAAGTTACAAAATCGCGCAGAGGC

42 GAATTATTCATTTCAATTACCTGATTGCGTAGATTTTCAGGTTTAAC

43 GTCAGATGAATATACAGTAACAGTCCTGATTGTTTGGATTATACTTC

44 TGAATAATGGAAGGGTTAGAACCAACAGTTAATGCCCCCTGCCT

45 ATTTCGGAACCTATTATTCTGTATCATCATATTCCTGATTATCAG

46 ATGATGGCAATTCATCAATATAACAAGTGTAGCGGTCACGCTGCGCGTAA

47 CCACCACACCCGCCGCGCTTAATGCGAACATTATCATTTTGCGGAACAAA

48 GAAACCACCAGAAGGAGCGGAATGAGGATTTAGAAGTATTAGACTTT

49 ACAAACAATTCGACAACTCGTATTGGCAAATCAACAGTTGAAAGGAA

50 TTGAGGAAGGTTATCTAAAATATTAGGAACCCATGTACCGTAACACTGAG

51 TTTCGTCACCAGTACAAACTACAACGCCTTGCTGAACCTCAAATATCAAA

52 CCCTCAATCAATATCTGGTCAGTACCAGCAGAAGATAAAACAGAGGT

53 GAGGCGGTCAGTATTAACACCGCGGCACAGACAATATTTTTGAATGG

54 CTATTAGTCTTTAATGCGCGAACAAAGGGATTTTAGACAGGAACGGTACG

55 CCAGAATCCTGAGAAGTGTTTTTATAGGCCAACAGAGATAGAACCCTTCT

56 GACCTGAAAGCGTAAGAATACGTAGTTGAGATTTAGGAATACCA

57 CATTCAACTAATGCAGATACAATTGGCAGATTCACCAGTCACACG

58 ACCAGTAATAAAAGGGACATTCTACAATATTACCGCCAGCCATTGCA

59 ACAGGAAAAACGCTCATGGAAATTTGCGGATGGCTTAGAGCTTAATTGCT

60 GAATATAATGCTGTAGCTCAACATGTCCTGAGTAGAAGAACTCAAACTAT

61 CGGCCTTGCTGGTAATATCCAGAATCAGTGAGGCCACCGAGTAAAAG

62 AGTCTGTCCATCACGCAAATTAATGCTTTCCTCGTTAGAATCAGAGC

63 GGGAGCTAAACAGGAGGCCGATTCCGCTACAGGGCGCGTACTATGGT

64 TGCTTTGACGAGCACGTATAACGGAGAAAGGAAGGGAAGAAAGCGAA

65 AGGAGCGGGCGCTAGGGCGCTGGTACCTTTTACATCGGGAGAAACAATAA

66 CGGATTCGCCTGATTGCTTTGAATACGGGAGCCCCCGATTTAGAGCTTGA

67 CGGGGAAAGCCGGCGAACGTGGCCCGTTGTAGCAATACTTCTTT

68 GATTAGTAATAACATCACTTGGTTTTTTGGGGTCGAGGTGCCGTA

69 AAGCACTAAATCGGAACCCTAAAAACAAGAGTCCACTATTAAAGAAC

70	GTGGACTCCAACGTCAAAGGGCGTCCTGTTTGATGGTGGTTCCGAAA
71	TCGGCAAAATCCCTTATAAATCAAGACGCTGAGAAGAGTCAATA
72	GTGAATTTATCAAAATCATAGAGAGTTGCAGCAAGCGGTCCACGC
73	TGGTTTGCCCCAGCAGGCGAAAATAGATACATTTCGCAAATGGTCAATAA
74	CCTGTTTAGCTATATTTTCATTTGGGCAGTGAGACGGGCAACAGCTGATT
75	GCCCTTCACCGCCTGGCCCTGAGAATGGTTTGAAATACCGACCG
76	TGTGATAAATAAGGCGTTAAAGGAGAGGCGGTTTGCGTATTGGGC
77	GCCAGGGTGGTTTTTCTTTTCACACTCACATTAATTGCGTTGCGCTC
78	ACTGCCCGCTTTCCAGTCGGGAACTGATAAATTGTGTCGAAATCCGCGAC
79	CTGCTCCATGTTACTTAGCCGGAACGGCCGGAAGCATAAAGTGTAAAGCC
80	TGGGGTGCCTAATGAGTGAGCTAGCGCGAGCTGAAAAGGTGGCATCA
81	ATTCTACTAATAGTAGTAGCATTAACAGTTGATTCCCAATTCTGCGA
82	ACGAGTAGATTTAGTTTGACCATAAAAACCGTCTATCAGGGCGATGGCCC
83	ACTACGTGAACCATCACCCAAATCAATTTAAATATGCAACTAAAGTACGG
84	TGTCTGGAAGTTTCATTCCATATAGGATTAGAGAGTACCTTTAATTG
85	CTCCTTTTGATAAGAGGTCATTTATTCGAGCTTCAAAGCGAACCAGA
86	CCGGAAGCAAACTCCAACAGGTCGTGTGAAATTGTTATCCGCTC
87	ACAATTCCACACAACATACGACAAAAAGATTAAGAGGAAGCCCGA
88	AAGACTTCAAATATCGCGTTTTACCCTCAAATGCTTTAAACAGTTCA
89	GAAAACGAGAATGACCATAAATCAAGAAGTTTTGCCAGAGGGGGTAA
90	TAGTAAAATGTTTAGACTGGATATAACGCCAAAAGGAATTACGAGGC
91	ATAGTAAGAGCAACACTATCATATAAAACGAACTAACGGAACAACAT
92	TATTACAGGTAGAAAGATTCATCCTGCAACAGTGCCACGCTGAGAGCCAG
93	CAGCAAATGAAAAATCTAAAGCATCATGGCTCATTATACCAGTCAGGACG
94	TTGGGAAGAAAAATCTACGTTAAAGAAACACCAGAACGAGTAGTAAA
95	TTGGGCTTGAGATGGTTTAATTTCTCCAAAAAAAAAGGCTCCAAA
96	AGGAGCCTTTAATTGTATCGGTCAACGTAACAAAGCTGCTCATTC
97	AGTGAATAAGGCTTGCCCTGACGACCCTCGTTTACCAGACGACGATAAAA
98	ACCAAAATAGCGAGAGGCTTTTGCAACTTCATCAAGAGTAATCTTGACAA
99	GAACCGGATATTCATTACCCAAACACCCTCAGCAGCGAAAGACA
100	GCATCGGAACGAGGGTAGCAAAGAGGACAGATGAACGGTGTACAG
101	ACCAGGCGCATAGGCTGGCTGACAAAAATCAGGTCTTTACCCTGACTATT
102	ATAGTCAGAAGCAAAGCGGATTGCATAGGCGCAGACGGTCAATCATAAGG
103	GAACCGAACTGACCAACTTTGAAATTATACCAAGCGCGAAACAAAGT
104	ACAACGGAGATTTGTATCATCGCAAAGAGGCAAAAGAATACACTAAA
105	ACACTCATCTTTGACCCCCAGCGCGGCTACAGAGGCTTTGAGGACTA
106	AAGACTTTTTCATGAGGAAGTTTGGTCGCTGAGGCTTGCAGGGAGTT
107	AAAGGCCGCTTTTGCGGGATCGTTTTATCAGCTTGCTTTCGAGGTGA

108	ATTTCTTAAACAGCTTGATACCGGGAACAACTAAAGGAATTGCGAAT
109	AATAATTTTTTCACGTTGAAAATATTTTGCTAAACAACTTTCAACAG
110	TTTCAGCGGAGTGAGAATAGAAAGCGAACCTCCCGACTTGCGGGAGGTTT
111	TGAAGCCTTAAATCAAGATTAGTTGCAGTTTTGTCGTCTTTCCAGACGTT
112	AGTAAATGAATTTTCTGTATGGGCAACTTTAATCATTGTGAATT
113	ACCTTATGCGATTTTAAGAACCCTGTAGCATTCCACAGACAGCCC
114	TCATAGTTAGCGTAACGATCTAAGCCACCCTCAGAGCCACCACCCTC
115	ATTTTCAGGGATAGCAAGCCCAAGGGTTGATATAAGTATAGCCCGGA
116	ATAGGTGTATCACCGTACTCAGGGGAAGCGCATTAGACGGGAGA
117	ATTAACTGAACACCCTGAACAGATTAGCGGGGTTTTGCTCAGTAC
118	CAGGCGGATAAGTGCCGTCGAGACTTTAGGAGCACTAACAACTAATAGAT
119	TAGAGCCGTCAATAGATAATACATTTAAACATGAAAGTATTAAGAGGCTG
120	AGACTCCTCAAGAGAAGGATTAGTAATAAGTTTTAACGGGGTCAGTG
121	CCTTGAGTAACAGTGCCCGTATAAAATAAATCCTCATTAAAGCCAGA
122	ATGGAAAGCGCAGTCTCTGAATTGAGCCGCCACCAGAACCACCACCA
123	GAGCCGCCGCCAGCATTGACAGGTAAATCAATATATGTGAGTGAATAACC
124	TTGCTTCTGTAAATCGTCGCTATTAATCAGAGCCGCCACCCTCAGAACCG
125	GTAATAAGAGAATATAAAGTAACCTGTCGTGCCAGCTGCATT
126	ACGTAAAACAGAAATAAAGAAAGGTTGAGGCAGGTCAGACGA
127	ATTAATTTTAAAAGTTTGAGTTGATAGCCCTAAAACATCGCC
128	ATTAAAAATACCGAACGAACCTAAATCCTTTGCCCGAACGTT
129	CGTCTGAAATGGATTATTTACGCGTCCAATACTGCGGAATCG
130	AATGAATCGGCCAACGCGCGGGGCAGAGGCATTTTCGAGCCA
131	TCATAAATATTCATTGAATCCACCTACATTTTGACGCTCAAT
132	TTGGCCTTGATATTCACAAACTACCATATCAAAATTATTTGC

Supplementary Table 3. Staple strands with sticky ends for DNA meshframe with symmetric valence modes. 2, 3, 4, 5 and 6 represent valence number. Color bars represent sequences used for each structure. These staples with sticky ends are used to replace the corresponding staples of DNA meshframe in Supplementary Table 2 (corresponding staple names on the left column) to construct DNA-encoded meshframe.

Staple name	Staples with sticky ends	2	3	4	5	6
87	ACAATTCCACACAACATACGACAAAAAGATTAAGAGGAAGCCCGAAACTAACTTCACA	■		■		■
59	ACAGGAAAAACGCTCATGGAAATTTGCGGATGGCTTAGAGCTTAATTGCTAACTAACTTCACA	■		■		■
58	ACCAGTAATAAAAGGGACATTCTACAATATTACCGCCAGCCATTGCAAACTAACTTCACA	■		■		■
88	AAGACTTCAAATATCGCGTTTTACCCTCAAATGCTTTAAACAGTTCAAACTAACTTCACA	■		■		■
85	CTCCTTTTGATAAGAGGTCATTTATTCGAGCTTCAAAGCGAACCAGAAACTAACTTCACA	■		■		■
131	TCATAAATATTCATTGAATCCACCTACATTTTGACGCTCAATAACTAACTTCACA	■		■		■
12	AATATTGACGGAAATTATTCATTTATAAAAGAAACGCAAAGACACCAAACTAACTTCACA	■		■		■
15	AACCGAGGAAACGCAATAATAACAATTGAGTTAAGCCCAATAATAAGAACTAACTTCACA	■		■		■
17	CTTTTGATGATACAGGAGTGTACTGGAAGTCAGAGGGTAATTGAGCGCTAAACTAACTTCACA	■		■		■
20	AGAAACGATTTTTTGTTTAACGTATCAATAGAAAATTCATATGGTTTACCAACTAACTTCACA	■		■		■
13	CGGAATAAGTTTATTTTGTCACAGGAATACCCAAAAGAACTGGCATGAACTAACTTCACA	■		■		■
18	ATATCAGAGAGATAACCCACAAGCAAAAATGAAAATAGCAGCCTTTAAACTAACTTCACA	■		■		■
35	TTACTAGAAAAAGCCTGTTTAGTACTTTTTCAAATATATTTTAGTTAAACTAACTTCACA		■	■	■	■
36	ATTTCATCTTCTGACCTAAATTTGTCTGAGAGACTACCTTTTTAACCAACTAACTTCACA		■	■	■	■
72	GTGAATTTATCAAAATCATAGAGAGTTGCAGCAAGCGGTCCACGCAACTAACTTCACA		■	■	■	■
74	CCTGTTTAGCTATATTTTCATTTGGGCAGTGAGACGGGCAACAGCTGATTAACTAACTTCACA		■	■	■	■
75	GCCCTTCACCGCCTGGCCCTGAGAATGGTTTGAAATACCGACCGAACTAACTTCACA		■	■	■	■
73	TCGGCAAAATCCCTTATAAATCAAGACGCTGAGAAGAGTCAATAAACTAACTTCACA		■	■	■	■
113	ACCTTATGCGATTTTAAGAACCCTGTAGCATTCCACAGACAGCCCAACTAACTTCACA			■		■
50	TTGAGGAAGGTTATCTAAAATATTAGGAACCCATGTACCGTAACACTGAGAACTAACTTCACA			■		■
92	TATTACAGGTAGAAAGATTCATCCTGCAACAGTGCCACGCTGAGAGCCAGAACTAACTTCACA			■		■
93	CAGCAAATGAAAAATCTAAAGCATCATGGCTCATTATACCAGTCAGGACGAACTAACTTCACA			■		■
112	AGTAAATGAATTTTCTGTATGGGCAACTTTAATCATTGTGAATTAACTAACTTCACA			■		■
51	TTTCGTCACCAGTACAAACTACAACGCCTTGCTGAACCTCAAATATCAAAAACTAACTTCACA			■		■
89	GAAAACGAGAATGACCATAAATCAAGAAGTTTTGCCAGAGGGGGTAAAACTAACTTCACA		■		■	
57	CATTCAACTAATGCAGATACAATTGGCAGATTCACCAGTCACACGAACTAACTTCACA		■		■	
90	TAGTAAAATGTTTAGACTGGATATAACGCCAAAAGGAATTACGAGGCAACTAACTTCACA		■		■	
56	GACCTGAAAGCGTAAGAATACGTAGTTGAGATTTAGGAATACCAAACTAACTTCACA		■		■	
129	CGTCTGAAATGGATTATTTACGCGTCCAATACTGCGGAATCGAACTAACTTCACA		■		■	
131	TCATAAATATTCATTGAATCCACCTACATTTTGACGCTCAATAACTAACTTCACA		■		■	
21	AGCGCCAAAGACAAAAGGGCGACATTCGAGCGTCTTTCCAGAGCCTAATTAACTAACTTCACA		■		■	

116	ATAGGTGTATCACCGTACTCAGGGGAAGCGCATTAGACGGGAGAAACTAACTTCACA
22	TGCCAGTTACAAAATAAACAGCCAGGTTTAGTACCGCCACCCTCAACTAACTTCACA
115	ATTTTCAGGGATAGCAAGCCCAAGGGTTGATATAAGTATAGCCCGGAAACTAACTTCACA
19	CAGAGAGAATAACATAAAAACAGATATTATTTATCCCAATCCAAATAAACTAACTTCACA
18	ATATCAGAGAGATAACCCACAAGCAAAAATGAAAATAGCAGCCTTTAAACTAACTTCACA
30	ACCATTACCATTAGCAAGGCCATGTTCAGCTAATGCAGAACGCGCAACTAACTTCACA
31	CTGTTTATCAACAATAGATAAGTCCATTAAACGGGTAAAATACGTAATGCAACTAACTTCACA
28	ATCGCCCACGCATAACCGATATATTCCCTGAACAAGAAAAATAATATCCCAACTAACTTCACA
105	ACACTCATCTTTGACCCCCAGCGCGGCTACAGAGGCTTTGAGGACTAAACTAACTTCACA
27	TCCTTATCATTCCAAGAACGGGTATAGTTGCGCCGACAATGACAACAACCAACTAACTTCACA
106	AAGACTTTTTCATGAGGAAGTTTGGTCGCTGAGGCTTGCAGGGAGTTAACTAACTTCACA
42	GAATTATTCATTTCAATTACCTGATTGCGTAGATTTTCAGGTTTAACAACTAACTTCACA
67	AGGAGCGGGCGCTAGGGCGCTGGTACCTTTTACATCGGGAGAAACAATAAAACTAACTTCACA
43	GTCAGATGAATATACAGTAACAGTCCTGATTGTTTGGATTATACTTCAACTAACTTCACA
66	TGCTTTGACGAGCACGTATAACGGAGAAAGGAAGGGAAGAAAGCGAAAACTAACTTCACA
45	ATTTCGGAACCTATTATTCTGTATCATCATATTCCTGATTATCAGAACTAACTTCACA
46	ATGATGGCAATTCATCAATATAACAAGTGTAGCGGTCACGCTGCGCGTAAAACTAACTTCACA

Supplementary Table 4. Staple strands with sticky ends for DNA meshframe with spherical helix valence mode. These staples with sticky ends are used to replace the corresponding staples of DNA meshframe in Supplementary Table 2 (corresponding staple names on the left column) to construct DNA-encoded meshframe.

Staple name	Staples with sticky ends
75	GCCCTTCACCGCCTGGCCCTGAGAATGGTTTGAAATACCGACCGAACTAACTTCACA
76	TGTGATAAATAAGGCGTTAAAGGAGAGGCGGTTTGCGTATTGGGCAACTAACTTCACA
34	TAACAACGCCAACATGTAATTTATAAGAATAAACACCGGAATCATAAAACTAACTTCACA
125	GTAATAAGAGAATATAAAGTAACCTGTCGTGCCAGCTGCATTAACTAACTTCACA
33	TCCAGACGACGACAATAAACAACGGCTTAATTGAGAATCGCCATATTAACTAACTTCACA
74	CCTGTTTAGCTATATTTTCATTTGGGCAGTGAGACGGGCAACAGCTGATTAAAGTCTCCTGA
73	TGGTTTGCCCCAGCAGGCGAAAATAGATACATTTCGCAAATGGTCAATAAAAAGTCTCCTGA
80	TGGGGTGCCTAATGAGTGAGCTAGCGCGAGCTGAAAAGGTGGCATCAAAAGTCTCCTGA
79	CTGCTCCATGTTACTTAGCCGGAACGGCCGGAAGCATAAAGTGTAAAGCCAAAGTCTCCTGA
77	GCCAGGGTGGTTTTTCTTTTCACACTCACATTAATTGCGTTGCGCTCAAAGTCTCCTGA
72	GTGAATTTATCAAAATCATAGAGAGTTGCAGCAAGCGGTCCACGCAACTAACTTCACA
70	GTGGACTCCAACGTCAAAGGGCGTCCTGTTTGATGGTGGTTCCGAAAAACTAACTTCACA
69	AAGCACTAAATCGGAACCCTAAAAACAAGAGTCCACTATTAAAGAACAACTAACTTCACA
82	ACGAGTAGATTTAGTTTGACCATAAAAACCGTCTATCAGGGCGATGGCCCAACTAACTTCACA
81	ATTCTACTAATAGTAGTAGCATTAACAGTTGATTCCCAATTCTGCGAAACTAACTTCACA
40	CATCAAGAAAACAAAATTAATTAAAAGAATAGCCCGAGATAGGGAAAGTCTCCTGA
41	TTGAGTGTTGTTCCAGTTTGGCAAGTTACAAAATCGCGCAGAGGCAAAGTCTCCTGA
65	AGGAGCGGGCGCTAGGGCGCTGGTACCTTTTACATCGGGAGAAACAATAAAAAGTCTCCTGA
66	CGGATTCGCCTGATTGCTTTGAATACGGGAGCCCCCGATTTAGAGCTTGAAAAGTCTCCTGA
68	GATTAGTAATAACATCACTTGGTTTTTTGGGGTCGAGGTGCCGTAAAAGTCTCCTGA
38	AAACATAGCGATAGCTTAGATTACATTTAACAATTTCATTTGAATTAAACTAACTTCACA
123	GAGCCGCCGCCAGCATTGACAGGTAAATCAATATATGTGAGTGAATAACCAACTAACTTCACA
122	ATGGAAAGCGCAGTCTCTGAATTGAGCCGCCACCAGAACCACCACCAAACTAACTTCACA
126	ACGTAAAACAGAAATAAAGAAAGGTTGAGGCAGGTCAGACGAAACTAACTTCACA
42	GAATTATTCATTTCAATTACCTGATTGCGTAGATTTTCAGGTTTAACAACTAACTTCACA
121	CCTTGAGTAACAGTGCCCGTATAAAATAAATCCTCATTAAAGCCAGAAAAGTCTCCTGA
16	AGCAAGAAACAATGAAATAGCAATACCGTTCCAGTAAGCGTCATACATGGAAAGTCTCCTGA
15	AACCGAGGAAACGCAATAATAACAATTGAGTTAAGCCCAATAATAAGAAAGTCTCCTGA
1	CCACCCTCAGAGCCACCACCCTCATAGCTATCTTACCGAAGCCCTAAAGTCTCCTGA
124	TTGCTTCTGTAAATCGTCGCTATTAATCAGAGCCGCCACCCTCAGAACCGAAAGTCTCCTGA
17	CTTTTGATGATACAGGAGTGTACTGGAAGTCAGAGGGTAATTGAGCGCTAAACTAACTTCACA
18	ATATCAGAGAGATAACCCACAAGCAAAAATGAAAATAGCAGCCTTTAAACTAACTTCACA

20	AGAAACGATTTTTTGTTTAACGTATCAATAGAAAATTCATATGGTTTACCAACTAACTTCACA
12	AATATTGACGGAAATTATTCATTTATAAAAGAAACGCAAAGACACCAAACTAACTTCACA
13	CGGAATAAGTTTATTTTGTCACAGGAATACCCAAAAGAACTGGCATGAACTAACTTCACA
21	AGCGCCAAAGACAAAAGGGCGACATTCGAGCGTCTTTCCAGAGCCTAATTAAAGTCTCCTGA
23	AGAACCGCCACCCTCAGAACCTATTTTGCACCCAGCTACAATTTTAAAGTCTCCTGA
24	ATCCTGAATCTTACCAACGCTAACAGATATAGAAGGCTTATCCGGTAAAAGTCTCCTGA
10	ACTTGAGCCATTTGGGAATTAGATTTCATCGTAGGAATCATTACAAAGTCTCCTGA
11	CGCGCCCAATAGCAAGCAAATCAACCGATTGAGGGAGGGAAGGTAAAAGTCTCCTGA
110	TTTCAGCGGAGTGAGAATAGAAAGCGAACCTCCCGACTTGCGGGAGGTTTAACTAACTTCACA
109	AATAATTTTTTCACGTTGAAAATATTTTGCTAAACAACTTTCAACAGAACTAACTTCACA
107	AAAGGCCGCTTTTGCGGGATCGTTTTATCAGCTTGCTTTCGAGGTGAAACTAACTTCACA
27	TCCTTATCATTCCAAGAACGGGTATAGTTGCGCCGACAATGACAACAACCAACTAACTTCACA
26	GAGAACAAGCAAGCCGTTTTTATAACCAATCAATAATCGGCTGTCTTAACTAACTTCACA
95	TTGGGCTTGAGATGGTTTAATTTCTCCAAAAAAAAGGCTCCAAAAAAGTCTCCTGA
96	AGGAGCCTTTAATTGTATCGGTCAACGTAACAAAGCTGCTCATTCAAAGTCTCCTGA
98	ACCAAAATAGCGAGAGGCTTTTGCAACTTCATCAAGAGTAATCTTGACAAAAAGTCTCCTGA
99	GAACCGGATATTCATTACCCAAACACCCTCAGCAGCGAAAGACAAAAGTCTCCTGA
106	AAGACTTTTTCATGAGGAAGTTTGGTCGCTGAGGCTTGCAGGGAGTTAAAGTCTCCTGA
90	TAGTAAAATGTTTAGACTGGATATAACGCCAAAAGGAATTACGAGGCAACTAACTTCACA
91	ATAGTAAGAGCAACACTATCATATAAAACGAACTAACGGAACAACATAACTAACTTCACA
93	CAGCAAATGAAAAATCTAAAGCATCATGGCTCATTATACCAGTCAGGACGAACTAACTTCACA
94	TTGGGAAGAAAAATCTACGTTAAAGAAACACCAGAACGAGTAGTAAAAACTAACTTCACA
97	AGTGAATAAGGCTTGCCCTGACGACCCTCGTTTACCAGACGACGATAAAAAACTAACTTCACA
56	GACCTGAAAGCGTAAGAATACGTAGTTGAGATTTAGGAATACCAAAAGTCTCCTGA
52	CCCTCAATCAATATCTGGTCAGTACCAGCAGAAGATAAAACAGAGGTAAAGTCTCCTGA
53	GAGGCGGTCAGTATTAACACCGCGGCACAGACAATATTTTTGAATGGAAAGTCTCCTGA
55	CCAGAATCCTGAGAAGTGTTTTTATAGGCCAACAGAGATAGAACCCTTCTAAAGTCTCCTGA
92	TATTACAGGTAGAAAGATTCATCCTGCAACAGTGCCACGCTGAGAGCCAGAAAGTCTCCTGA
48	GAAACCACCAGAAGGAGCGGAATGAGGATTTAGAAGTATTAGACTTTAACTAACTTCACA
49	ACAAACAATTCGACAACTCGTATTGGCAAATCAACAGTTGAAAGGAAAACTAACTTCACA
51	TTTCGTCACCAGTACAAACTACAACGCCTTGCTGAACCTCAAATATCAAAAACTAACTTCACA
127	ATTAATTTTAAAAGTTTGAGTTGATAGCCCTAAAACATCGCCAACTAACTTCACA
128	ATTAAAAATACCGAACGAACCTAAATCCTTTGCCCGAACGTTAACTAACTTCACA

Supplementary Table 5. Staple strands with sticky ends for DNA meshframe with polychromatic valence. 1, 2, 3, 4 and 5 represent DNA meshframe for $P_1(345)P_3(12)$, $P_1(245)P_4(13)$, $P_1(345)P_2(2)P_3(1)$, $P_1(245)P_2(3)P_3(1)$ and $P_1(24)P_2(3)P_3(15)$, respectively. Color bars represent sequences used for each structure. These staples with sticky ends are used to replace the corresponding staples of DNA meshframe in Supplementary Table 2 (corresponding staple names on the left column) to construct DNA-encoded meshframe.

Staple name	Staples with sticky ends	1	2	3	4	5
30	ACCATTACCATTAGCAAGGCCATGTTCAGCTAATGCAGAACGCGCAAAGTCTCCTGA	■	■	■	■	■
31	CTGTTTATCAACAATAGATAAGTCCATTAAACGGGTAAAATACGTAATGCAAAGTCTCCTGA	■	■	■	■	■
28	ATCGCCCACGCATAACCGATATATTCCCTGAACAAGAAAAATAATATCCCAAAGTCTCCTGA	■	■	■	■	■
105	ACACTCATCTTTGACCCCCAGCGCGGCTACAGAGGCTTTGAGGACTAAAGTCTCCTGA	■	■	■	■	■
27	TCCTTATCATTCCAAGAACGGGTATAGTTGCGCCGACAATGACAACAACCAAAGTCTCCTGA	■	■	■	■	■
106	AAGACTTTTTCATGAGGAAGTTTGGTCGCTGAGGCTTGCAGGGAGTTAAAGTCTCCTGA	■	■	■	■	■
42	GAATTATTCATTTCAATTACCTGATTGCGTAGATTTTCAGGTTTAACAACTAACTTCACA		■		■	■
67	AGGAGCGGGCGCTAGGGCGCTGGTACCTTTTACATCGGGAGAAACAATAAAACTAACTTCACA		■		■	■
43	GTCAGATGAATATACAGTAACAGTCCTGATTGTTTGGATTATACTTCAACTAACTTCACA		■		■	■
66	TGCTTTGACGAGCACGTATAACGGAGAAAGGAAGGGAAGAAAGCGAAAACTAACTTCACA		■		■	■
45	ATTTCGGAACCTATTATTCTGTATCATCATATTCCTGATTATCAGAACTAACTTCACA		■		■	■
46	ATGATGGCAATTCATCAATATAACAAGTGTAGCGGTCACGCTGCGCGTAAAACTAACTTCACA		■		■	■
42	GAATTATTCATTTCAATTACCTGATTGCGTAGATTTTCAGGTTTAACAAAGTCTCCTGA	■				
67	AGGAGCGGGCGCTAGGGCGCTGGTACCTTTTACATCGGGAGAAACAATAAAAAGTCTCCTGA	■				
43	GTCAGATGAATATACAGTAACAGTCCTGATTGTTTGGATTATACTTCAAAGTCTCCTGA	■				
66	TGCTTTGACGAGCACGTATAACGGAGAAAGGAAGGGAAGAAAGCGAAAAAGTCTCCTGA	■				
45	ATTTCGGAACCTATTATTCTGTATCATCATATTCCTGATTATCAGAAAGTCTCCTGA	■				
46	ATGATGGCAATTCATCAATATAACAAGTGTAGCGGTCACGCTGCGCGTAAAAAGTCTCCTGA	■				
42	GAATTATTCATTTCAATTACCTGATTGCGTAGATTTTCAGGTTTAACAAGAACCTGCTA			■		
67	AGGAGCGGGCGCTAGGGCGCTGGTACCTTTTACATCGGGAGAAACAATAAAAGAACCTGCTA			■		
43	GTCAGATGAATATACAGTAACAGTCCTGATTGTTTGGATTATACTTCAAGAACCTGCTA			■		
66	TGCTTTGACGAGCACGTATAACGGAGAAAGGAAGGGAAGAAAGCGAAAAGAACCTGCTA			■		
45	ATTTCGGAACCTATTATTCTGTATCATCATATTCCTGATTATCAGAAGAACCTGCTA			■		
46	ATGATGGCAATTCATCAATATAACAAGTGTAGCGGTCACGCTGCGCGTAAAAGAACCTGCTA			■		
89	GAAAACGAGAATGACCATAAATCAAGAAGTTTTGCCAGAGGGGGTAAAACTAACTTCACA	■	■	■		
57	CATTCAACTAATGCAGATACAATTGGCAGATTCACCAGTCACACGAACTAACTTCACA	■	■	■		
90	TAGTAAAATGTTTAGACTGGATATAACGCCAAAAGGAATTACGAGGCAACTAACTTCACA	■	■	■		
56	GACCTGAAAGCGTAAGAATACGTAGTTGAGATTTAGGAATACCAAACTAACTTCACA	■	■	■		
129	CGTCTGAAATGGATTATTTACGCGTCCAATACTGCGGAATCGAACTAACTTCACA	■	■	■		
131	TCATAAATATTCATTGAATCCACCTACATTTTGACGCTCAATAACTAACTTCACA	■	■	■		

89	GAAAACGAGAATGACCATAAATCAAGAAGTTTTGCCAGAGGGGGTAAAAGAACCTGCTA
57	CATTCAACTAATGCAGATACAATTGGCAGATTCACCAGTCACACGAAGAACCTGCTA
90	TAGTAAAATGTTTAGACTGGATATAACGCCAAAAGGAATTACGAGGCAAGAACCTGCTA
56	GACCTGAAAGCGTAAGAATACGTAGTTGAGATTTAGGAATACCAAAGAACCTGCTA
129	CGTCTGAAATGGATTATTTACGCGTCCAATACTGCGGAATCGAAGAACCTGCTA
131	TCATAAATATTCATTGAATCCACCTACATTTTGACGCTCAATAAGAACCTGCTA
35	TTACTAGAAAAAGCCTGTTTAGTACTTTTTCAAATATATTTTAGTTAAACTAACTTCACA
36	ATTTCATCTTCTGACCTAAATTTGTCTGAGAGACTACCTTTTTAACCAACTAACTTCACA
72	GTGAATTTATCAAAATCATAGAGAGTTGCAGCAAGCGGTCCACGCAACTAACTTCACA
74	CCTGTTTAGCTATATTTTCATTTGGGCAGTGAGACGGGCAACAGCTGATTAACTAACTTCACA
75	GCCCTTCACCGCCTGGCCCTGAGAATGGTTTGAAATACCGACCGAACTAACTTCACA
73	TCGGCAAAATCCCTTATAAATCAAGACGCTGAGAAGAGTCAATAAACTAACTTCACA
21	AGCGCCAAAGACAAAAGGGCGACATTCGAGCGTCTTTCCAGAGCCTAATTAACTAACTTCACA
116	ATAGGTGTATCACCGTACTCAGGGGAAGCGCATTAGACGGGAGAAACTAACTTCACA
22	TGCCAGTTACAAAATAAACAGCCAGGTTTAGTACCGCCACCCTCAACTAACTTCACA
115	ATTTTCAGGGATAGCAAGCCCAAGGGTTGATATAAGTATAGCCCGGAAACTAACTTCACA
19	CAGAGAGAATAACATAAAAACAGATATTATTTATCCCAATCCAAATAAACTAACTTCACA
18	ATATCAGAGAGATAACCCACAAGCAAAAATGAAAATAGCAGCCTTTAAACTAACTTCACA
21	AGCGCCAAAGACAAAAGGGCGACATTCGAGCGTCTTTCCAGAGCCTAATTAAAGTCTCCTGA
116	ATAGGTGTATCACCGTACTCAGGGGAAGCGCATTAGACGGGAGAAAAGTCTCCTGA
22	TGCCAGTTACAAAATAAACAGCCAGGTTTAGTACCGCCACCCTCAAAGTCTCCTGA
115	ATTTTCAGGGATAGCAAGCCCAAGGGTTGATATAAGTATAGCCCGGAAAAGTCTCCTGA
19	CAGAGAGAATAACATAAAAACAGATATTATTTATCCCAATCCAAATAAAAGTCTCCTGA
18	ATATCAGAGAGATAACCCACAAGCAAAAATGAAAATAGCAGCCTTTAAAAGTCTCCTGA

Supplementary Table 6. Thiolated DNA strands for AuNP modification.

Name	Sequence	Modified AuNPs
S1	HS-C_6H_{12}-TTTTTAGTAGTGTGAAGTTAG	NPs in symmetric clusters, NPs on odd sites of spherical helix cluster, P_1 in multi-type clusters
S2	HS-C_6H_{12}-TTTTTTTTTTTTTTTTCAGGAGACT	NPs on even sites of spherical helix cluster, P_3 in multi-type clusters, P_4 in P_1(245)P_4(13)
S3	HS-C_6H_{12}-TTTTTTTTTTTTTTTTAGCAGGTTC	P_2 in multi-type clusters

Supplementary References

1. Benson, E., Mohammed, A., Gardell, J., Masich, S., Czeizler, E., Orponen, P.*, et al.* DNA rendering of polyhedral meshes at the nanoscale. *Nature* **523,** 441-444 (2015).

2. Tian, C., Cordeiro, M. A. L., Lhermitte, J., Xin, H. L., Shani, L., Liu, M.*, et al.* Supra-nanoparticle functional assemblies through programmable stacking. *ACS Nano* **11,** 7036-7048 (2017).

3. Draine, B. T., Flatau, P. J. Discrete-dipole approximation for scattering calculations. *J. Opt. Soc. Am. A* **11,** 1491-1499 (1994).
